# Loss of a Neural AMP-Activated Kinase Mimics the Effects of Elevated Serotonin on Fat, Movement, and Hormonal Secretions

**DOI:** 10.1371/journal.pgen.1004394

**Published:** 2014-06-12

**Authors:** Katherine A. Cunningham, Aude D. Bouagnon, Alexandre G. Barros, Lin Lin, Leandro Malard, Marco Aurélio Romano-Silva, Kaveh Ashrafi

**Affiliations:** 1 Department of Physiology, University of California, San Francisco, San Francisco, California, United States of America; 2 Instituto Nacional de Ciência e Tecnologia de Medicina Molecular, Faculdade de Medicina da Universidade Federal de Minas Gerais, Belo Horizonte, Brazil; 3 Departamento de Física, Instituto de Ciências Exatas da Universidade Federal de Minas Gerais, Belo Horizonte, Brazil; Stanford University, United States of America

## Abstract

AMP-activated protein kinase (AMPK) is an evolutionarily conserved master regulator of metabolism and a therapeutic target in type 2 diabetes. As an energy sensor, AMPK activity is responsive to both metabolic inputs, for instance the ratio of AMP to ATP, and numerous hormonal cues. As in mammals, each of two genes, *aak-1* and *aak-2*, encode for the catalytic subunit of AMPK in *C. elegans*. Here we show that in *C. elegans* loss of *aak-2* mimics the effects of elevated serotonin signaling on fat reduction, slowed movement, and promoting exit from dauer arrest. Reconstitution of *aak-2* in only the nervous system restored wild type fat levels and movement rate to *aak-2* mutants and reconstitution in only the ASI neurons was sufficient to significantly restore dauer maintenance to the mutant animals. As in elevated serotonin signaling, inactivation of AAK-2 in the ASI neurons caused enhanced secretion of dense core vesicles from these neurons. The ASI neurons are the site of production of the DAF-7 TGF-β ligand and the DAF-28 insulin, both of which are secreted by dense core vesicles and play critical roles in whether animals stay in dauer or undergo reproductive development. These findings show that elevated levels of serotonin promote enhanced secretions of systemic regulators of pro-growth and differentiation pathways through inactivation of AAK-2. As such, AMPK is not only a recipient of hormonal signals but can also be an upstream regulator. Our data suggest that some of the physiological phenotypes previously attributed to peripheral AAK-2 activity on metabolic targets may instead be due to the role of this kinase in neural serotonin signaling.

## Author Summary

While it is well appreciated that food availability has profound effects on behavior, physiology, and metabolism, the molecular systems that link these complex processes together still remain poorly understood. An ancient cellular sensor of energy is AMP-activated protein kinase, AMPK. Here we show that in the genetically tractable *C. elegans*, loss of AMPK in the nervous system mimics many of the outcomes also seen upon elevated serotonin signaling, a neural indicator of food availability. We show that similar to elevated serotonin signaling, loss of neural AMPK causes reduced movement while enhancing fat metabolism and secretions of neuroendocrine hormones known to be systemic regulators of energy balance, development and aging. While AMPK is generally considered a mediator of hormonal signaling, our findings indicate that it also regulates their release. Our findings suggest that some previous results attributed to roles of AMPK in the regulation of peripheral metabolism may in fact be due to the roles of this kinase complex in the nervous system as a mediator of serotonin signaling.

## Introduction

AMP-activated protein kinase (AMPK) is a sensor of energy status that is conserved from single celled yeasts to humans [1]. At the cellular level, it becomes activated in response to deficits in energy availability, such as a rise in the ratio of AMP to ATP, to inhibit energy utilizing pathways while activating energy generating pathways [1]. AMPK is also a recipient of many hormonal signals and links organism-wide signals of energy balance with myriad cellular mechanisms that are differentially regulated based on energy availability [2]. While many of the consequences of AMPK activity are due to its regulation of substrates in peripheral tissues, activity of hypothalamic AMPK in mammals is also thought to contribute to energy balance through modulation of feeding behavior [3]. Given its broad effects on energy balance, AMPK is a therapeutic target for type 2 diabetes as well as certain cancers [4], [5]. Despite the key role of this kinase complex in energy balance and its therapeutic relevance, many of the physiological consequences of AMPK activity and its upstream inputs and downstream effectors still remain poorly understood.

*C. elegans* provides a genetically tractable system for studying the physiological roles of AMPK in the context of whole animals. In *C. elegans* as in mammals, AMPK is a kinase complex with catalytic α and regulatory β and γ subunits [1], [6]. Similar to mammals, two genes, *aak-1* and *aak-2*, separately encode AMPK's catalytic α subunit [6]. Thus far, roles for *aak-2* have been reported in several facets of *C. elegans* biology including the regulation of feeding [7], fat [1], [8], [9], L1 diapause and nutrient deprivation [1], [10], dauer maintenance [2], [9], [11], [12], and longevity [3], [6], [13], [14]. In most of these processes, the requirement for *aak-2* has been attributed to its roles in peripheral tissues.

As in mammals, 5- hydroxytryptamine (5-HT), serotonin, signaling in *C. elegans* serves as an indicator of food availability [4], [5], [15], [16]. Animals deficient in 5-HT signaling due to inactivation of tryptophan hydroxylase, *tph-1*, the rate limiting enzyme in serotonin biosynthesis, exhibit many of the phenotypes seen when animals are removed from food [1], [6], [15]. In turn, treatment of animals with exogenous serotonin elicits many of the phenotypes seen when food deprived animals are re-exposed to food [6], [17]–[20]. Beyond simply an indication of food availability, serotonin signaling informs on a number of other inputs, including food quality [7], [21], [22], pathogenicity [22], [23], and the experience of starvation prior to re-feeding [18], [19], [24]. For instance, when taken off food, *C. elegans* quickly reduce their feeding, as measured by the pharyngeal pumping rate [18], and once again elevate it as they are reintroduced to food. If animals experience a period of fasting, they display an even greater increase in feeding rate upon re-encountering food [18]. This increased feeding rate can also be induced if well-fed animals, which already have elevated levels of serotonin signaling relative to food deprived animals, are treated with serotonin or fluoxetine, a serotonin uptake inhibitor [25]. In another example, as animals deplete their food stores, they move more rapidly, presumably to forage for new food resources, and slow their movement once they find such resources [17]. If animals are food-deprived for a period of time before they re-encounter food, they exhibit a more significant slowing of movement known as enhanced slowing. This enhanced slowing is dependent on serotonin signaling since animals deficient in serotonin production only partially exhibit the enhanced slowing response and addition of serotonin to well-fed animals reduces movement rate [17], [26]. Thus, serotonin is not simply a binary, on/off indicator of food availability but levels of serotonin signaling allow animals to tune their behavioral and physiological responses to food related cues and experiences.

We previously found that 5-HT secreted from the ADF neurons, a pair of ciliated sensory neurons that are known to be responsive to the environment [27], acts on the SER-5 serotonergic receptor on the AVJ pair of interneurons to modulate food intake rate [7]. Our studies suggested that signaling through SER-5, a G_αs_ coupled receptor, leads to activation of protein kinase A (PKA) and subsequent PKA-mediated phosphorylation of AAK-2 at residue S244 causing inhibition of AAK-2 activity, likely through inhibition of phosphorylation at the adjacent T243 residue, equivalent of T172 in mammalian AMPK catalytic subunit [7], [28]. Thus, loss of AAK-2 activity mimicked the very high levels of feeding seen food deprived animals encounter food or well-fed animals are treated with additional doses of serotonin.

Here, we demonstrate that loss of *aak-2* mimics the effects of elevated serotonin signaling on enhanced fat metabolism, reduced movement, and exit from the dauer state. In the context of dauer maintenance, we show that inactivation of *aak-2* links serotonin signaling to the release of DAF-7, a TGF-β family ligand, and DAF-28, encoding an insulin family member, from the ciliated ASI neurons in a cell autonomous fashion. While AMPK is often considered a downstream effector of hormonal signaling, our results show that AAK-2 containing AMPK complexes are also upstream regulators. Importantly, by revealing that serotonin signaling exerts many of its effects on animal behavior and physiology by inactivation of AAK-2 containing AMPK complexes in the nervous system, our data suggest a need for re-interpretation of some of the findings in which the requirement for *aak-2* had only been attributed to its peripheral, metabolic roles.

## Results

### Loss of *aak-2* mimics effects of elevated serotonin signaling on movement and fat

**Figure 1:**
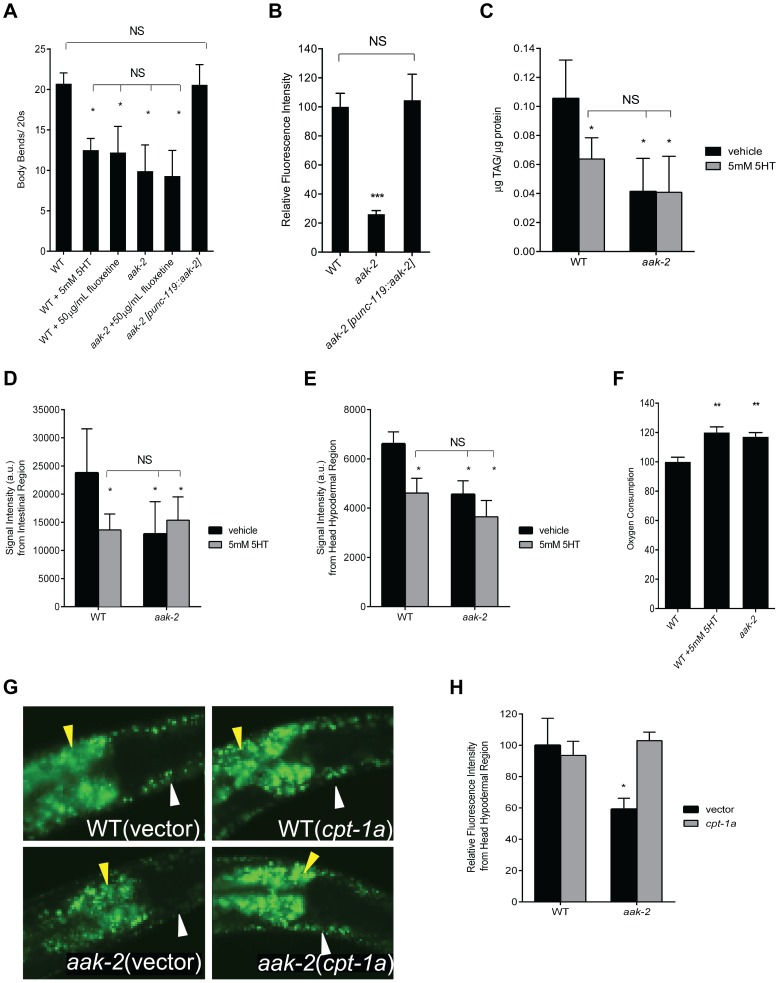
Loss of *aak-2* mimics increased serotonin signaling. **A.** *aak-2* mutants and wild type animals (WT) treated with exogenous serotonin (5 mM) or fluoxetine (50 µg/mL) have fewer body bends when removed from food. Pan-neuronal reconstitution of *aak-2* (p*unc-119*) restores wild type body bends to *aak-2* mutants. n = 10, *p<0.05, one-way ANOVA with Bonferroni correction for multiple comparisons. **B.** *aak-2* deficient animals have reduced hypodermal BODIPY fluorescence relative to WT. Wild type BODIPY staining is restored to *aak-2* mutants when *aak-2* is reconstituted in the nervous system (p*unc-119*). n = 10, ***p<0.001, one-way ANOVA with Bonferroni correction for multiple comparisons. **C.** Wild type animals treated with 5 mM 5-HT have significantly lower triglycerides per protein (TAG/protein) compared to sham treatment as determined by total lipid extraction followed by Thin Layer Chromatography. Sham treated *aak-2* mutants already have significantly lower TAG/protein compared to WT and 5 mM 5-HT treatment does not result in further reduction. TAG/protein levels of 5-HT treated WT are not significantly different than those of *aak-2* +/− 5-HT. n = 3, *p<0.05, Student's t-test. **D-E.** Quantitation of signal intensities of Coherent anti-Stokes Raman Scattering, CARS, of WT and *aak-2* mutants +/− 5 mM 5-HT treatment. 5-HT treatment lowered the CARS signal intensities from the intestinal (D) and head hypodermal (E) regions of WT animals. *aak-2* mutants had lower signal intensities relative to WT, which was not further reduced by 5 mM 5-HT treatment. Signal intensities of 5-HT treated WT were not significantly different than those of *aak-2* +/− 5-HT. n = 5, *p<0.01, Student's t-test. **F.** Loss of *aak-2* or 5 mM 5-HT treatment caused elevated oxygen consumption. WT and *aak-*2 mutants (n = 800 per genotype) were sham treated. Data are expressed as a percentage of WT. Error bars represented +/− SEM. **p < 0.01 versus sham treated WT, one-way ANOVA with Bonferroni correction for multiple comparisons **G-H.** Loss of *cpt-1a* via RNAi restores wild type BODIPY staining to hypodermis (white arrow) and intestine (yellow arrow) of *aak-2* mutants. Representative BODIPY staining images (G) and corresponding quantitations (H) are shown. n = 5, *p<0.05, Student's t-test.

Given that serotonin exerted its effects on feeding in part through inactivation of AAK-2 containing AMPK complexes, we asked whether *aak-2* mutants exhibit any other phenotypic consequences of elevated serotonin signaling. Consistent with the enhanced slowing response, *aak-2* deficient animals had reduced movement rate off-food (Figure 1A; [28]). The rate of movement of *aak-2* mutant animals was similar to wild type animals exposed to the serotonin reuptake inhibitor, fluoxetine, and the already slowed off-food movement of *aak-2* mutants was not further reduced by fluoxetine treatment (Figure 1A).

To determine the relationship between exogenous serotonin treatment and the effects of endogenous serotonin increase by fluoxetine treatment, we conducted dose response studies. As previously reported [26], we noted that transient treatment of wild type animals off of food with increasing doses of exogenous 5-HT causes a progressive slowing of movement culminating in sickness and paralysis (Figure S1A-B). Based on the dose-response studies and consistent with previous similar studies [20], [29], [30], we chose the 5 mM exogenous 5-HT concentration as one that mimics the movement phenotypes of wild type animals treated with high doses of fluoxetine (Figure S1A). While both wild type and *aak-*2 deficient animals became paralyzed with increasing doses of exogenous serotonin, *aak-2* mutants were more sensitive to paralyzing effects of high doses of serotonin, consistent with the notion that these mutants already experience elevated serotonin signaling (Figure S1B). Using pharyngeal pumping rate as another read-out, we found that treatment of wild type animals with 5 mM 5-HT caused elevated feeding rate similar to fluoxetine treatment and increasing the dose of serotonin to 10 mM did not cause further feeding elevation (Figure S1C). The already elevated feeding rate of *aak-2* deficient animals was unchanged with increasing doses of exogenous serotonin levels up 10 mM. However, as in the case of movement, *aak-2* mutants were more sensitive to deleterious effects of elevated 5-HT, such that elevating the dose of exogenous 5-HT beyond 10 mM had a feeding reducing effect accompanied by other signs of sickness (Figure S1C).

As in mammals, enhanced serotonergic signaling in *C. elegans* causes fat reduction [19]. While in mammals, the fat reducing effects of serotonin have been attributed to its anorectic effects, in *C. elegans* serotonergic regulation of feeding behavior is through a cellular circuit that is distinct from serotonergic regulation of fat metabolism [7], [19]. The fat reducing effects of elevated serotonin in *C. elegans* are due to enhancement of fat utilization in peripheral tissues. Recent studies have suggested that a complex regulatory loop between serotonin and octopamine signaling cascades in the nervous system ultimately leads to transcriptional upregulation of various components of fat mobilization and oxidation machineries in the periphery [19], [31].

To determine whether loss of *aak-2* mimics the effects of elevated serotonin on fat, we assessed fat content using the fluorescent BODIPY-labeled fatty acids and biochemical measurement of triacylglycerides, both of which indicated that *aak-2* mutants had reduced fat compared to wild type (Figure 1B-C). The reduced fat phenotype of *aak-2* mutants was similar in magnitude to wild type animals subjected to 5 mM exogenous 5-HT and not further diminished upon 5-HT treatment (Figure 1C-E; [19]). To verify these results, we used Coherent anti-Stokes Raman Scattering, CARS, a label free microscopic method for the assessment of fat levels [32]–[34]. The CARS results corroborated the noted fat reduction upon *aak-2* inactivation and 5 mM serotonin treatment (Figure 1D-E; [19]). Moreover, the notion that *aak-2* mutants have particularly low fat levels in their skin-like hypodermal tissues, a result readily suggested by treatment of these mutants with BODIPY-labeled fatty acids (Figure 1B), was corroborated by CARS (Figure 1E).

Fat reducing effects of serotonin elevation are associated with increased total oxygen consumption [19]. Accordingly, *aak-2* mutants exhibited elevated rates of oxygen consumption relative to wild type animals (Figure 1F). We next examined transcriptional expression patterns of nearly a hundred fat and sugar metabolic genes [35], [36] by RT-PCR. Although the precise magnitude of changes were not identical and there were genes that were differentially regulated in *aak-*2 mutants and serotonin treated wild type animals (Figure S1D, Table S1), we noted a significant overlap between those that were upregulated, downregulated, or unchanged in *aak-*2 mutants and wild type animals treated with 5 mM 5-HT compared to untreated wild type animals (Figure S1D, Table S1). Genes that were significantly upregulated by both elevated serotonin and *aak-2* inactivations included homologs of an acyl-CoA synthase, required for activation of fatty acids, a carnitine palmitoyl transferase, which can shuttle fatty acids across the mitochondrial membranes for fat oxidation, and a mitochondrial acyl-CoA dehydrogenase, an enzymatic component of fat oxidation. The similarities in the patterns of transcriptional changes in various metabolic genes supported the notion that elevated serotonin and loss of *aak-2* affect fat metabolism through a common pathway. Finally, we examined the effects of inactivations of W01A11.5, encoding a putative carnitine palmitoyl transferase, *cpt-1a*, and F08A8.4, encoding a putative acyl-CoA oxidase, on fat content of *aak-2* mutants. This is because we had previously shown that these gene inactivations partially block the fat reducing effects of elevated serotonin signaling [19]. These gene inactivations also partially blocked the fat reducing effects of *aak-2* deficiency (Figure 1G-H, Figure S2A-C).

These results combined with our previous findings suggested that loss of *aak-2* mimics the effects of elevated serotonin signaling on food intake behavior, fat, and movement.

### Effects of losses of *aak-1* and *tph-1* on *aak-2* mutant phenotypes

**Figure 2:**
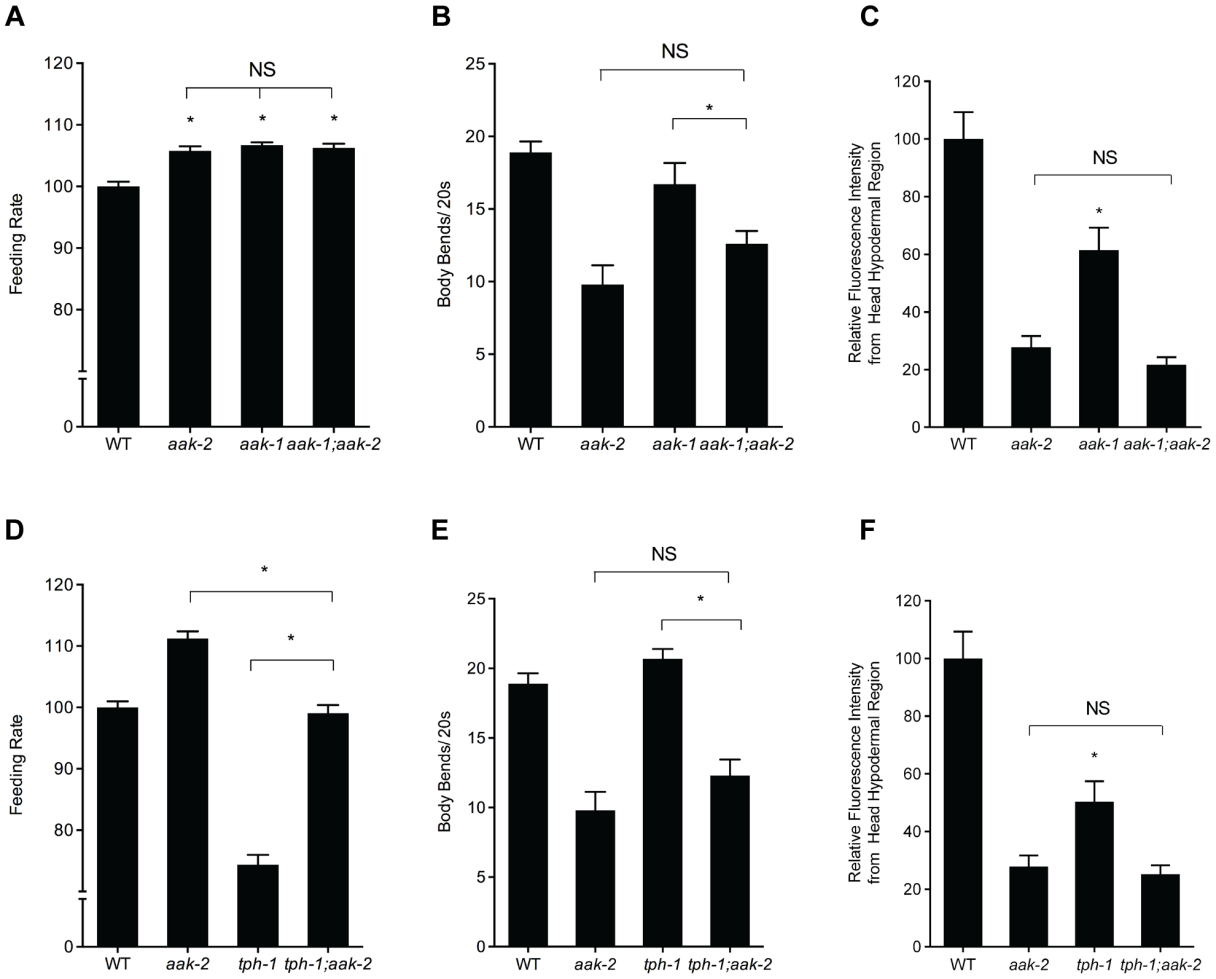
Effects of losses of *aak-1* and *tph-1* on *aak-2* mutant phenotypes. **A-C.** Phenotypes of *aak-2* loss of function are not dependent on *aak-1*. Feeding (A), movement (B), and hypodermal BODIPY staining levels (C) of *aak-1; aak-2* double mutants are not significantly different than those of *aak-2* mutants. For feeding, movement and BODIPY measurements, n = 10, *p<0.05, Student's t-test. Error bars represent +/−SEM. **D-F.** Loss of *aak-2* elicits feeding (D), movement (E), and fat phenotypes (**F**) even in serotonin deficient *tph-1* mutants. The feeding rate of *tph-1; aak-2* mutants was significantly different than both *tph-1* and *aak-2* single mutants (D). *tph-1; aak-2* double mutants moved significantly more slowly than *tph-1* or WT but statistically indistinguishable than *aak-2* mutants off of food (E) For BODIPY staining in the hypodermal head region, loss *aak-2* further reduced the already low hypodermal head staining of *tph-1* mutants (F). For feeding, movement and BODIPY measurements, n = 10, *p<0.05, Student's t-test. Error bars represent +/−SEM. Please note that the BODIPY quantitations are of the head hypodermal region only. While *tph-1* mutants have been reported to have elevated intestinal fat levels, their head hypodermal region actually has less staining relative to WT animals. To be consistent with our various other BODIPY measurements, we have concentrated on the same head hypodermal region when comparing *tph-1* with *tph-1; aak-2.*

As both *aak-1* and *aak-2* separately encode for the catalytic subunits of AMPK. We considered the possibility that the phenotypes caused by loss of *aak-2* may in fact be due to aberrant activation of *aak-1*. However, none of the phenotypes of *aak-2* mutants were altered in *aak-1; aak-2* double mutants (Figure 2A-C). Additionally, *aak-1* transcript levels were unchanged in *aak-2* mutants (Figure S3). Thus, the phenotypes caused by loss of *aak-2* were not simply compensatory responses of AAK-1 activation.

To decipher the relationship between serotonin production and *aak-2*, we next examined *tph-1; aak-2* double mutants. The feeding, fat, and movement phenotypes of the double mutants were either the same as those of *aak-2* mutants or intermediate between those of *tph-1* and *aak-2* single mutants (Figure 2D-F). One interpretation of these results is that *aak-2* functions, at least in part, upstream of *tph-1*. For instance, loss of *aak-2* could promote production of serotonin by elevating *tph-1* expression. However, expression of *tph-1* was unchanged in *aak-2* mutants relative to wild type animals (Figure S3). A second interpretation is that *aak-2* functions downstream of *tph-1* but only mediates some of the effects of serotonin on fat, feeding, and movement. Since we previously found that in the context of feeding regulation, elevated serotonin acts through the SER-5 receptor to cause inhibition of AAK-2 in interneurons that are not known to produce serotonin, we favor the second interpretation. This interpretation is consistent with the notion that loss of *aak-2* mimics phenotypes seen when serotonin signaling is elevated beyond that of steady state well-fed animals.

### Reconstitution of *aak-2* in the nervous system restores wild type fat and movement to *aak-2* mutants

In *C. elegans*, *aak-2* is broadly expressed in both neural and peripheral tissues [9], [28]. To determine whether loss of *aak-2* in specific tissues could account for its movement and fat phenotypes, we reconstituted wild type *aak-2* in select tissues of the mutant animals. Reconstitution of *aak-*2 throughout the nervous system restored wild type movement rate (Figure 1A) and fat levels to *aak-2* mutants (Figure 1B) while reconstitutions within body wall muscle, intestine, pharyngeal muscle, and hypodermis failed to do so (Figure S4 and data not shown).

We previously reported that normalized feeding rate is restored when *aak-2* is reconstituted within only the AVJ pair of interneurons as well as the pharynx of *aak-2* mutants [7]. These transgenic animals, however, still exhibited reduced fat levels that were indistinguishable from those of *aak-2* mutants (Figure S4). These data suggested that elevated serotonin signaling elicits feeding and fat phenotypes through inhibition of AAK-2 containing complexes in distinct regions of the nervous system.

### Loss of *aak-2* causes fat reduction independent of the dauer state

The observations that loss of neural *aak-2* mimics the effects of serotonin signaling forced us to re-evaluate previously published claims where requirements for *aak-2* in various physiological processes have been largely attributed to its peripheral, metabolic roles. One such example is the requirement for *aak-2* in fat rationing during the dauer stage [9]. The dauer stage of *C. elegans* is an altered developmental state that is restricted to an early larval stage [37]. Entry into the dauer state is initiated by lack of nutrients or excessive population density, which lead to reductions in activities of pro-growth and developmental pathways of insulin signaling and TGF-β signaling [37]. Relative to non-dauer early larval animals, dauers contain elevated lipid levels, which presumably acts as an energetic reservoir for these non-feeding animals [37].

**Figure 3:**
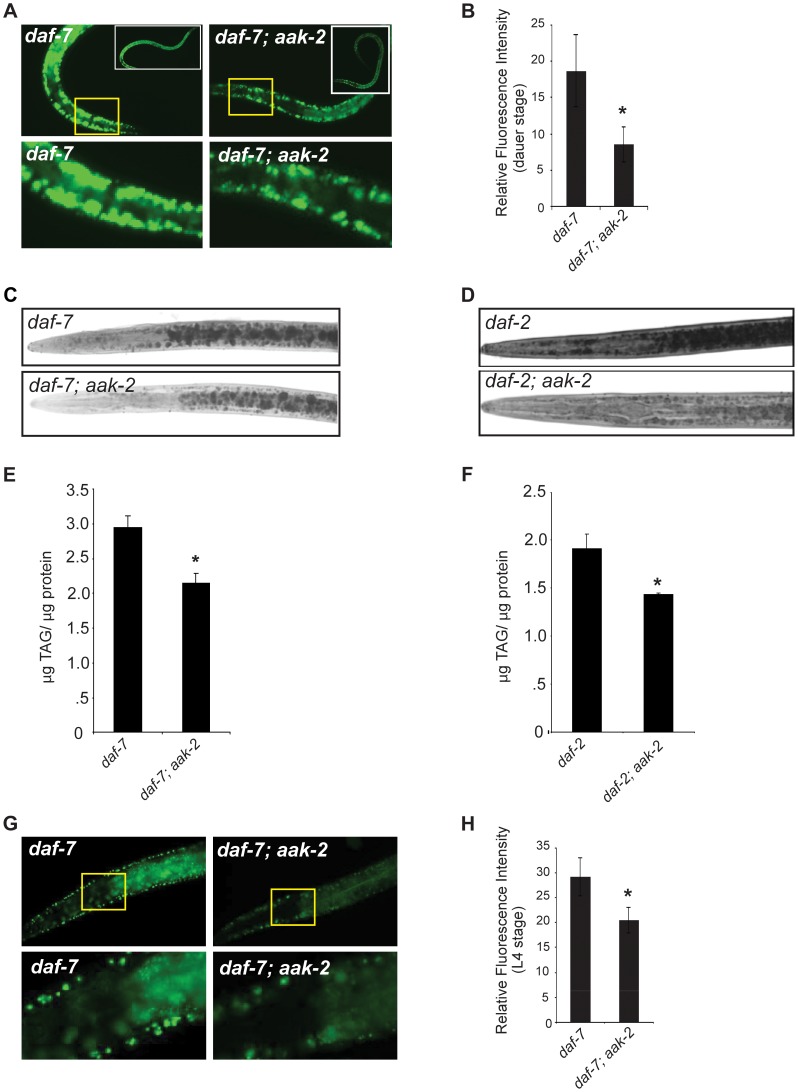
*daf-7; aak-2* and *daf-2; aak-2* have reduced fat relative to *daf-7* and *daf-2* at all stages of development. **A.** *daf-7; aak-2* dauers have significantly reduced BODIPY fluorescence relative to *daf-7* on the first day after the dauer molt. Insets show entire animals and the bottom panel shows a zoomed-in selection, denoted by the yellow box, from the top panel. **B.** Quantification of anterior hypodermal BODIPY fluorescence for *daf-7* and *daf-7; aak-2* animals on the first day after the dauer molt. n = 8. * p<0.05, Student's t-test. **C.** Representative Sudan Black B staining of *daf-7* (top) or *daf-7; aak-2* (bottom) animals on the first day after the dauer molt. **D.** Representative Sudan Black B staining of *daf-2* (top) or *daf-2; aak-2* (bottom) animals on the first day after the dauer molt. **E.** *daf-7; aak-2* animals have significantly lower TAG/protein than *daf-7* animals kept at the restrictive temperature (25°C) on the first day after the dauer molt as determined by total lipid extraction followed by Thin Layer Chromatography. n = 4, * p<0.05, Student's t-test. **F.** *daf-2; aak-2* animals have significantly lower TAG/protein than *daf-2* animals kept at the restrictive temperature (25°C) on the first day after the dauer molt as determined by total lipid extraction followed by Thin Layer Chromatography. n = 4, * p<0.05, Student's t-test. **G.** L4 stage *daf-7; aak-2* animals have significantly reduced BODIPY fluorescence intensity relative to *daf-7* animals. Insets show the anterior portion of the animal and the bottom panel shows a zoomed-in selection from the top panel. **H.** Quantification of anterior hypodermal BODIPY fluorescence for *daf-7* and *daf-7; aak-2* animals as L4 animals. n = 10, * p<0.05, Student's t-test. Error bars represent +/-SEM.

A previous study suggested that *aak-2* is required for proper fat rationing during the dauer state [9]. Animals deficient in *daf-2,* encoding an insulin receptor-like gene, or in *daf-7,* encoding a neurally expressed TGF-β family ligand that links environmental conditions to growth and development pathways, enter the dauer state constitutively [38], [39]. It was previously reported that *daf-2; aak-2* and *daf-7; aak-2* double mutants enter the dauer state with the expected high levels of lipids but then suffer a rapid fat depletion during the dauer state, implicating a specific role for AAK-2 in lipid rationing during the dauer stage [9]. This contrasted with our finding that the low fat phenotype of *aak-*2 deficient animals was not restricted to the dauer stage. To explore this potential discrepancy, we re-examined fat contents of *daf-7; aak-*2 and *daf-2; aak-2* double mutants [9]. Multiple independent methods of assessing fat levels — vital staining with BODIPY-labeled fatty acids, fixed staining with Sudan Black B, and total lipid extraction followed by thin layer chromatography — all showed significant reductions in lipid levels in *daf-7; aak-2* and *daf-2; aak-2* relative to *daf-7* and *daf-2* mutants, respectively, both prior to and at the time of dauer entry (Figure 3, S5). We also examined fat levels of *daf-2; aak-2* double mutants with CARS, and consistent with all of other results, noted that these double mutants had low fat levels (Figure S6). Moreover, the CARS experiments indicated that treatment of *daf-2* animals with 5 mM exogenous serotonin causes fat reductions to the same extent as that seen in *daf-2; aak-2* mutants and exogenous serotonin treatment did not further reduce the fat content of these double mutants (Figure S6). Thus, the role of *aak-2* in fat regulation is not dauer stage-specific and the low fat phenotype of *aak-2* deficient animals is not dependent on either intact insulin or TGF-β pathways.

### *aak-2* deficient animals exit dauer even in the absence of food cues

Under normal physiological circumstances, dauers must be able to sense the reappearance of food to resume growth. Serotonin, an indicator of food availability, is known to promote dauer exit [15]. Therefore, we considered the possibility that the noted requirement for *aak-2* in dauer survival may reflect an increased tendency of animals to exit the dauer stage rather than a failure to maintain dauer survival due to rapid exhaustion of lipid stored during this state.

The claim that *aak-*2 dauers fail to maintain survival has been based on studies that rely on a “dauer trap” system, where dauers are kept suspended within a sterile water drop and their survival is periodically assessed within the drop [9], [11], [12]. We noted that at the time of dauer entry nearly 100% of *daf-2; aak-2* and *daf-7; aak-2* double mutants as well as *daf-2* or *daf-7* single mutants survived 1% SDS treatment, a treatment traditionally used to distinguish dauers from non-dauers [40] (data not shown). Thus, at least by this criteria *aak-2* deficient animals enter the dauer stage. After a few days in the dauer trap assay, *aak-2* deficient animals exhibited features associated with animals that have exited dauer yet fail to grow due to lack of nutrients. These features included loss of radial constriction and growth of the germ-line as well as behaviors such as pumping that are never seen in dauers (data not shown). These findings suggested that rather than dying as dauers, *aak-2* deficient animals appear to exit the dauer state but then starve to death under the conditions of the dauer trap.

**Figure 4:**
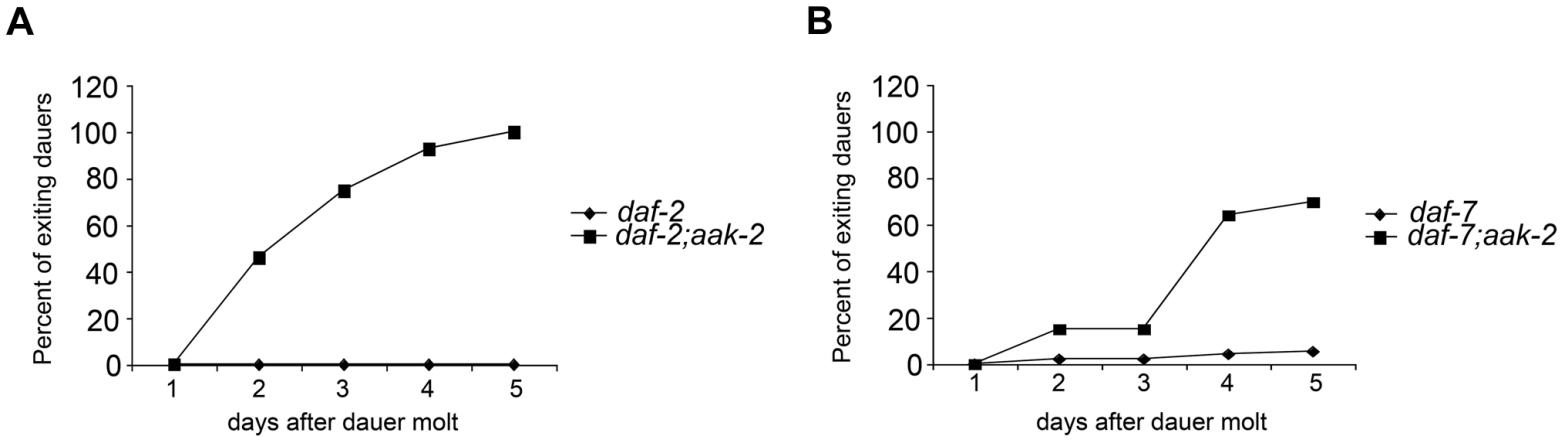
*aak-*2 deficiency causes dauer exit. **A-B.** 100% *daf-2; aak-2* (A) and *daf-7; aak-2* (B) animals exit dauer when kept on plates with food (*E. coli* OP50) at the restrictive temperature (25°C) and resume reproductive, while 100% *daf-2* (A) and *daf-*7(B) mutants animals maintain dauer under similar conditions. A representative comparison is shown. Similar rates of exit and growth were found in at least 5 independent experiments per comparison.

To distinguish the possibility that *aak-2* deficient animals expire as dauers or prematurely exit this stage, we examined *daf-2; aak-2* and *daf-7; aak-2* double mutants when maintained as dauers on plates with food (*E. coli*) or within the dauer trap assay also supplemented with food. In a timeframe coincident with previously reported loss of viability during the dauer state, the *aak-2* deficient animals exited the dauer state and grew into reproductive adults (Figure 4A-B). We also rarely observed any *aak-2* deficient animals that died as dauers. These findings suggest that the reported requirements for *aak-2* in proper dauer survival actually reflect increased tendency of these animals to exit this stage and are consistent with the notion that loss of *aak-2* elicits a set of phenotypes also seen upon enhanced serotonin signaling.

### Loss of *aak-2* mimics 5-HT signaling in promoting neuroendocrine secretions from the ASI neurons

**Figure 5:**
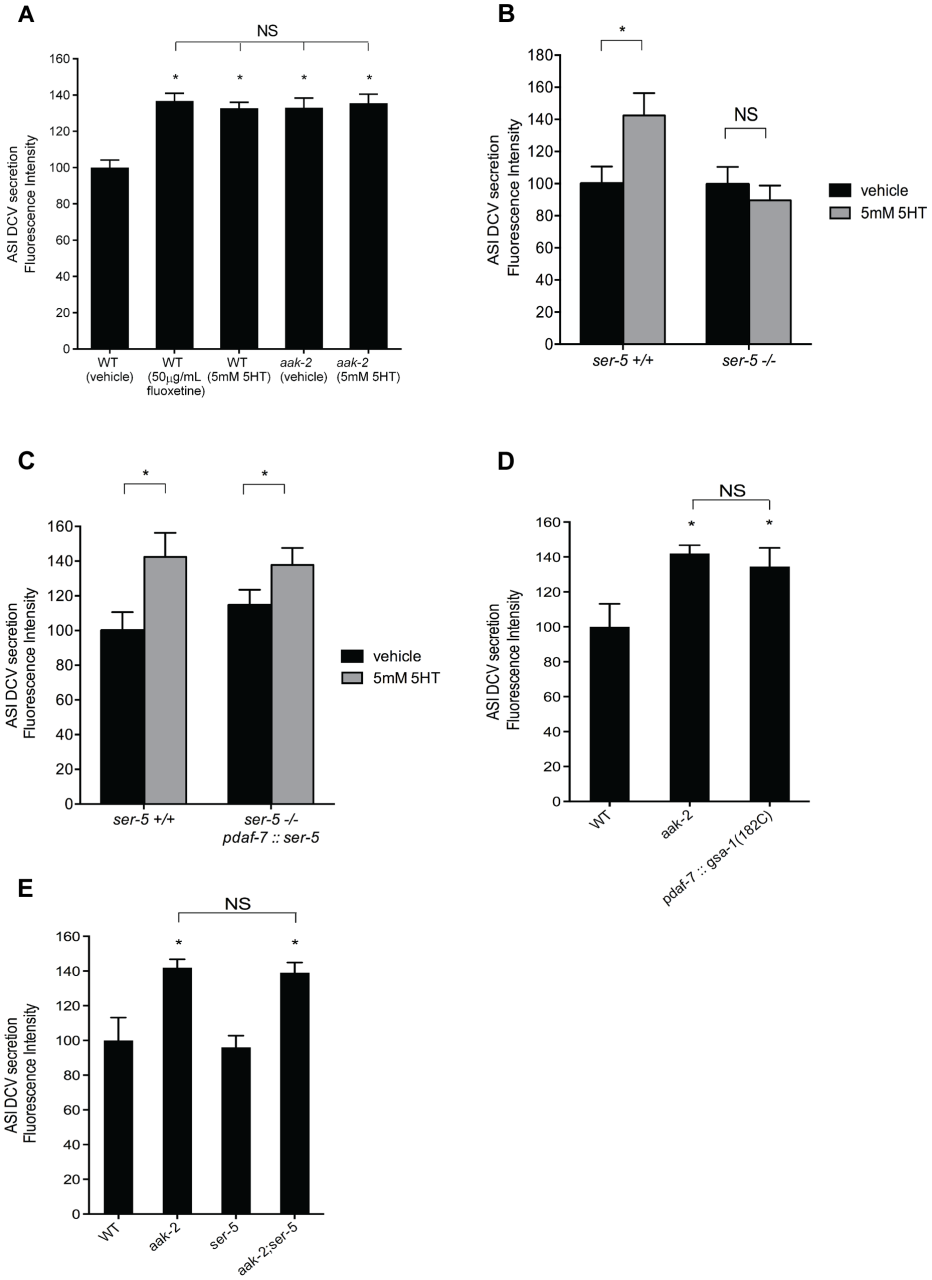
Elevated serotonin or loss of *aak-2* cause increased dense-core vesicle secretion from ASI neurons. **A-B.** Quantitation of tagged DAF-28::mCherry (A) and DAF-7::mCherry (B) accumulation in coelomocytes. *daf-28::mCherry* and *daf-7::mCherry* were expressed in the ASI neurons using a *daf-7* promoter. Error bars represent standard error. * p<0.05 relative to WT, Student's t-test **C.** Quantitation of tagged DAF-28::mCherry accumulation in coelomocytes when *ser-5* is reconstituted in ASI (p*daf-7*) in otherwise *ser-5* deficient animals. * p<0.05 relative to WT, Student's t-test. **D.** Secretion of DAF-28::mCherry from the ASI neurons is elevated upon ASI specific expression of *gsa-1*(R182C), encoding a gain-of-function version of G_αs_, previously shown to cause inactivation of AAK-2. Error bars represent standard error. * p<0.05, Student's t-test, relative to WT **E.** Elevated secretion of DAF-28::mCherry form ASI in *aak-2* mutants is not dependent on *ser-5.* Error bars represent standard error. * p<0.05 relative to WT, Student's t-test. In A-E, p*unc-122*::*GFP* was used to mark coelomocytes. Each bar represents examination of 20–30 transgenic animals. For each comparison, the transgene of interest was introduced into indicated backgrounds by crossing.

Serotonin is thought to promote enhanced signaling through the insulin and TGF-β signaling pathways in *C. elegans* [16]. Therefore, we asked whether inactivation of AAK-2 links serotonin signaling to these signaling cascades. The ciliated sensory neurons ASI secrete the DAF-7 TGF-β ligand as well as DAF-28, an insulin-like peptide, both of which are packaged and secreted in dense-core vesicles [41], [42]. To assess the effects of serotonin and *aak-2* on dense-core vesicle secretion, we fused full-length DAF-28 to a fluorescent mCherry reporter and expressed the fusion in ASI neurons using the *daf-7* promoter [43], [44]. Such transgenes have been used to quantitatively assess dense-core vesicle mediated secretions from various *C. elegans* neurons including the ASI [41], [45], [46]. Secreted peptides accumulate within coelomocytes, scavenger cells that non-specifically endocytose molecules within the pseudocoelom. This reporter system has been validated by a variety of assays that have functionally probed the consequences of dense core vesicle secretions [45]–[47]. Treatment of wild type animals with exogenous 5-HT resulted in a ∼30% increase of fluorescent signal accumulation in coelomocytes (Figure 5A). Similarly, loss of *aak-2* increased accumulation of the DAF-28 reporter, which was not further enhanced by exogenous serotonin treatment (Figure 5A). Similar results were obtained when examining coelomocyte accumulation of a full length DAF-7 fused to mCherry and expressed in the ASI neurons (Figure S7A).

We next set out to determine whether the enhanced coelomocyte accumulation of DAF-28 and DAF-7 fusion reporters in *aak-2* mutants were due to increased transcriptional activity, enhanced secretion, or both. Transcriptional expression levels of *daf-7* and *daf-28* were indistinguishable in wild type and *aak-2* mutants as assessed by RT-PCR assays (Figure S7B). To examine whether the enhanced accumulation of the reporter fusions in the coelomocytes depended on dense core vesicle secretions, we generated *aak-2; unc-31* double mutants expressing the DAF-28::mCherry transgenic secretion reporter. *unc-31* is a neurally expressed gene that encodes for the *C. elegans* homolog of Calcium Activated Protein for Secretion, CAPS, which is critical for fusion of dense core vesicles with plasma membranes [48]. Loss of *unc-31* is known to block enhanced secretion of dense core vesicles in *C. elegans* [41]. Not only was the elevated coelomocyte accumulations of DAF-28::mCherry of *aak-2* mutants abrogated by loss of *unc-31*, the levels were below those seen in wild type animals and similar to the levels of *unc-31* mutants (Figure S7C). These findings suggested that the enhanced secretions seen in *aak-2* mutants require the canonical dense core vesicle release machinery.

Within the context of feeding regulation, we previously found that 5-HT acts through the G protein-coupled receptor, SER-5, to ultimately cause inactivation of AAK-2 in the AVJ pair of interneurons [7]. As *ser-5* is expressed in the ASI neurons, we tested whether it links serotonin to enhanced secretions from these neurons. Indeed, loss of *ser-5* abrogated enhanced accumulation of reporter fusions secreted from the ASI upon serotonin treatment (Figure 5B). In turn, selective reconstitution of *ser-5* only in the ASI neurons of *ser-5* mutants once again allowed for serotonin induced secretion from these neurons (Figure 5C). Additionally, selective ASI expression of *gsa-1*(R182C), encoding a gain-of-function mutation in G_αs_ [49], expected to mimic enhanced signaling through the G_αs_ coupled SER-5 receptor [50] and causing inactivation of AAK-2 [7], led to increased dense-core vesicle secretion from the ASI neurons (Figure 5D). Finally, we showed that *aak-2; ser-5* double mutants had increased ASI secretions similar to those seen in *aak-2* mutants (Figure 5E) and that loss of *aak-2* did not alter *ser-5* gene expression (Figure S7B).

The epistasis and cell specific reconstitution studies presented above suggest that serotonin promotes enhanced secretions from the ASI neurons through activation of SER-5 and subsequent inactivation of AAK-2 in the ASI neurons to promote enhanced secretion of DAF-7 TGF-β and the DAF-28, which play critical roles in whether animals stay in dauer or undergo reproductive growth.

### Increased neuroendocrine signals contribute to dauer exit of *aak-2* mutants

**Figure 6:**
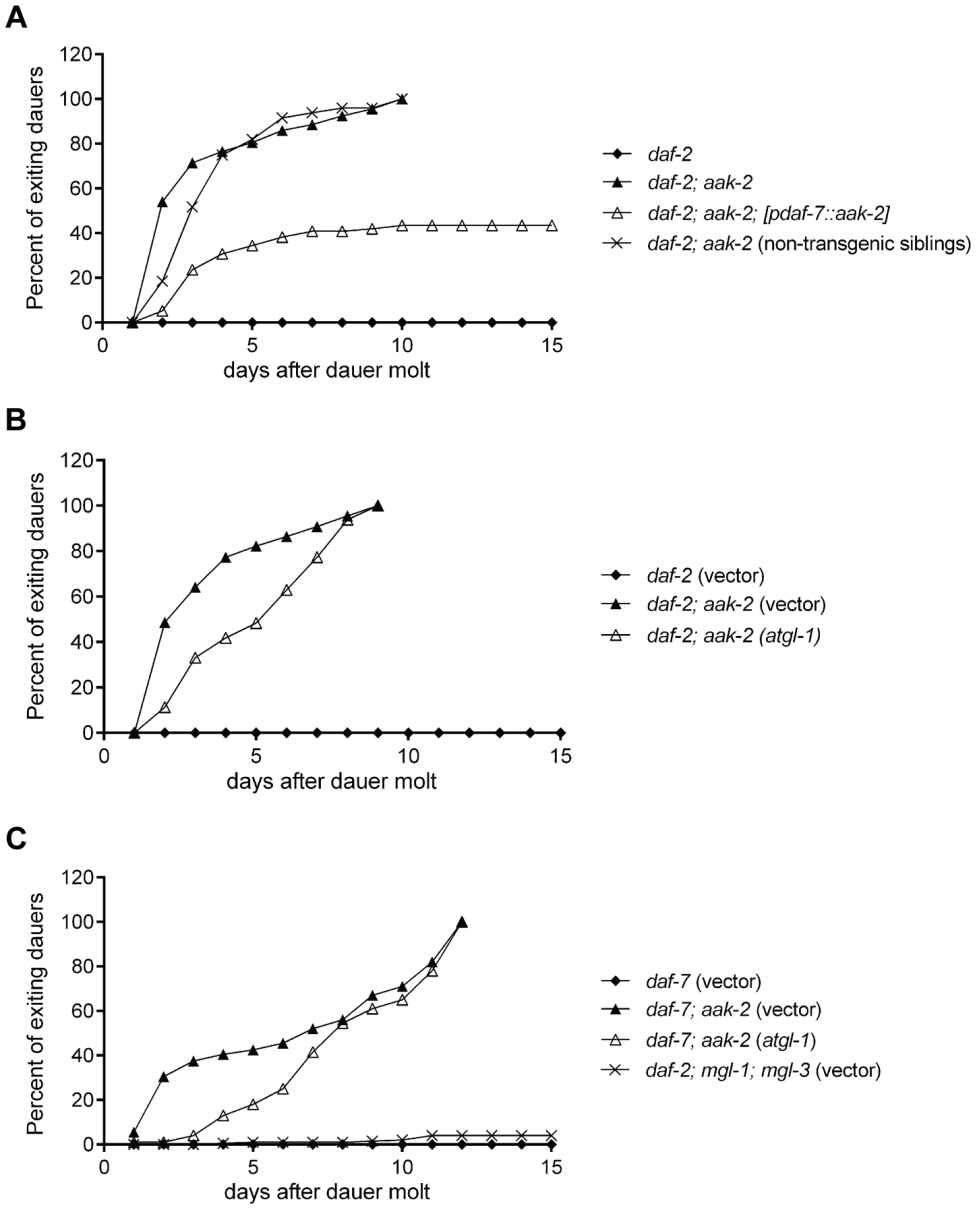
Reconstitution of *aak-2* in ASI partially rescues dauer exit. **A.** *daf-2; aak-2* animals exit dauer when kept on plates at the restrictive temperature (25 °C), while *daf-*2 animals maintain dauer. Reconstitution of *aak-2* in only the ASI neurons restores dauer maintenance to ∼60% of *daf-2; aak-2* double mutants. Non-transgenic siblings do not show any improved dauer maintenance. Animals exiting dauers grew to adulthood in each case. We did not examine these dauers beyond day 15 only because it became increasingly difficult to maintain these plates contamination free and prevent the dauers from escaping the plates. **B-C.** RNAi-mediate knockdown of *atgl-1* delays dauer exit for *daf-2; aak-2* (B) and *daf-7; aak-2* (C) animals but does not allow for dauer maintenance beyond the time that a 100% of vector treated animals have exited the stage and resumed growth. While relative to *daf-7* mutants, *daf-7; mgl-1; mgl-3* triple mutants have low fat levels, they maintain dauer as well as *daf-7* mutants (C). Each of the graphed data in A-C reflects the averages of 5 plates of 100 animals per genotype. A representative result is shown. The indicated results were repeated in at least three independent trials.

Elevated secretions of TGF-β like ligand, DAF-7, and insulins, are known to counteract the dauer constitutive phenotypes of *daf-2* and *daf-7* mutants [38], [39]. We reasoned that if enhanced DAF-7 secretion from the ASI neurons accounted, at least in part, for tendency of *daf-2; aak-2* mutant animals to exit the dauer stage, then reconstitution of *aak-2* in ASI neurons should promote maintenance of the dauer state. This was indeed the case. While *daf-2* mutants stayed in dauer throughout the course of the experiment, by 8 days, virtually all *daf-2; aak-2* had exited dauer. By contrast, after 8 days, ∼60% of transgenic animals in which *aak-2* was only reconstituted in the ASI neurons, were still in the dauer state (Figure 6A). They retained viability as dauers since they remained responsive to a gentle touch (data not shown). All of these animals remained in the dauer state until the experiment was terminated on day 15.

It was previously reported that loss of *atgl-1,* encoding a lipase required for mobilization of triglycerides [51], allowed dauer survival in *aak-2* deficient animals by preventing fat loss of these mutants [9]. We therefore wondered whether the ability of the subset of *daf-2; aak-2* deficient animals in which *aak-2* was reconstituted in the ASI neurons could be due to restoration of fat levels. This was, however, not the case since the fat levels of these animals were indistinguishable from that of *daf-2; aak-2* mutants (Figure S4).

Our dauer maintenance assays were done in the presence of food such that we could distinguish animals that exit the dauer stage from those that might expire as dauers. We re-assessed the role *atgl-1* loss in preventing dauer exit using our assay conditions. Under these conditions, 100% of *daf-2* or *daf-7* mutants remained in the dauer state by day 10 post dauer entry, while nearly 100% of *daf-7; aak-2* or *daf-2; aak-2* mutants had exited the dauer and resumed growth. Loss of *atgl-1* delayed the rate of dauer exit especially during days 2–7, however, by day 10 still nearly 100% of animals had exited the dauer stage and resumed growth (Figure 6B). To further investigate the contribution of fat levels on dauer maintenance, we screened through a metabolic sub-library of RNAi clones to identify additional genes that are important for fat reduction when *aak-2* is lost. We identified several peroxisomal genes whose inactivation increased BODIPY-labeled fatty acid staining in *daf-7; aak-2* animals: *pmp-1, pmp-2, daf-22*, and *prx-5* (data not shown). Like *atgl-1*, loss of these peroxisomal genes caused a 2-3 day delay in the time frame of dauer exit but did not change the finding that nearly a 100% of animals existed the dauer stage by day 10 (data not shown). Finally, we examined dauer maintenance of *daf-7; mgl-1; mgl-3* triple mutants. This is because losses of the neurally expressed metabotropic glutamate receptors encoded by *mgl-1* and *mgl-3* significantly reduce fat levels of *daf-7* mutants [52]. While *daf-7* mutants have nearly 2.5 fold more lipid staining based on Sudan Black B compared *daf-7; mgl-1; mgl-3* triple mutants [52], the three triple mutants maintained dauer survival virtually similar to *daf-7* mutants (Figure 6C). Thus, increasing lipid reserve causes a modest delay in dauer exit but does not prevent it. Similarly, reduction of lipid reservoirs is insufficient to promote dauer exit in *daf-7* mutants.

## Discussion

In *C. elegans*, serotonin signaling modulates a series of food related behavioral, physiological, and metabolic responses. We previously showed that elevated serotonin signaling leads to an increase in feeding rate through inhibition of AAK-2 containing AMPK complexes in the nervous system. The data presented here demonstrate that inactivation of *aak-2* also mimics the effects of elevated serotonin on fat reduction, reduced movement, and whether animals stay in dauer or undergo reproductive growth and development. In the context of dauer decision, our data are consistent with a model whereby serotonin signaling through the SER-5 receptor leads to inactivation of AAK-2 in the ASI neurons, in turn, promoting enhanced release of the DAF-7 TGF-β ligand and insulins from these neurons (Figure S8).

Although reconstitution of *aak-2* in only the nervous system of animals was sufficient to revert many of the phenotypes of *aak-*2 animals to nearly wild type levels, the requirement for *aak-2* activity mapped to different regions of the nervous system for various phenotypes of *aak-2* mutants. For instance, reconstitution of *aak-2* in only the *hlh-34* expression neurons plus the pharynx of *C. elegans* was sufficient to restore wild type feeding rates but did not alter the fat, movement, or dauer maintenance phenotypes of *aak-2* mutants. In turn, restoration of *aak-2* to the ASI neurons of *C. elegans*, the site of production of the TGF-β ligand DAF-7, was sufficient to restore normalized secretion of dense-core vesicles from these neurons and significantly restored dauer maintenance without restoration of wild type feeding, fat, or movement to the *aak-2* mutants. We do not yet know which subset of serotonin responsive neurons may specifically account for the fat and movement effects caused by loss of *aak-2*. The finding that serotonergic regulation of feeding, fat, movement, and dauer exit through inhibition of AAK-2 occur in different regions of the nervous system indicates that behavioral and physiological processes that broadly regulate energy balance, while coordinated by serotonin signaling, are not simply consequences of one another and can be differentially modulated by the nervous system.

The recognition that loss of *aak-2* mimics the effects of serotonin signaling forced us to re-evaluate some of the interpretations of the physiological roles attributed to AAK-2. Specifically, it had been suggested that *aak-2* deficient dauers fail to inhibit proliferation of their germ-lines and fail to maintain survival during this stage due to i) a failure in rationing of lipid reservoirs, ii) inappropriate osmotic regulation, and iii) lack of a hormesis-like effect caused by inappropriately high catalase activity [11]. In each of these cases, the dauer trap assay was used to monitor survival of dauers [9], [11]. We found that this assay makes it difficult to distinguish between animals that exit dauer and succumb to early death due to factors such as starvation and animals that fail to maintain survival while remaining in the dauer stage. This distinction is important for appropriately understanding the role of *aak-2* in dauer physiology. For instance, *aak-2* mutants that have been maintained in the dauer trap assay for several days exhibit lower fat levels, an outcome that has been interpreted as a failure by these animals to ration their lipid reserves during the dauer state [9], [11]. However, lower lipid levels would also be expected if *aak-2* deficient dauers exit this stage and resume growth but then starve due to lack of nutrient availability in the dauer trap assay. To differentiate early dauer exit from death during the dauer state, we added *E. coli* to the dauer trap assay or kept the *aak-2* dauers on plates with food. Under these circumstances, we did not see *aak-2* mutants that expired as dauers. Rather, we found that virtually all *aak-*2 deficient dauers exited this state and resumed normal patterns of growth. Moreover, we found that elevation of serotonin signaling or loss of *aak-2* promoted enhanced release of the DAF-7 TGF-β and the DAF-28 insulin, systemic regulators of animal growth and development. Thus, our data suggest that the noted effects of loss of *aak-2* on dauer survival are due its requirement for preventing early dauer exit. Our results, however, neither rule out a role for AAK-2 in modulating metabolism in the periphery including during periods of nutrient deprivation, nor challenge the notion that AAK-2 may regulate the activity of ATGL-1. In fact, a recent study indicated that fat reduction induced by elevated serotonin signaling in the nervous system, a condition that we suggest is mimicked by loss of *aak-2* from the nervous system, depends on transcriptional upregulation of *atgl-1* in the periphery [31].

Our analyses of fat staining in *aak-2* mutants led to different conclusions than those previously reported for these mutants [9]. These discrepant results highlight some of the methodological challenges in assessing *C. elegans* fat levels. Fixed staining and biochemical methods were previously used to claim that *aak-2* mutants enter dauer with wild type levels of fat [9]. Using the same methodologies, we instead found that *aak-2* mutants have lower levels of fat at all stages including at the time of dauer entry. The results of our fixed dye and biochemical fat measurements were corroborated by vital BODIPY-fatty acid labeling as well as label free CARS. Although fixed staining methods and biochemical methods have in the past few years been touted as the strategies by which *C. elegans* fat should be assessed [53], [54], in our experience both methods are prone to an enormous amount of variability and can be fairly insensitive when used to gauge total lipid contents of whole animals. Fixed staining methods rely on permeabilization of the cuticle to allow penetrance of dyes, a process that can be difficult to achieve uniformly. The fixed staining methods also rely on alcohol dehydration steps, which if not done properly can dissolve away triglycerides. Similarly, the biochemical measurements of extracted triglycerides are prone to a great deal of experimental variation since they rely on relatively large populations of animals and extraction procedures that can have vastly different efficiencies in different trials. Thus, while each of these methods can provide valid assessments of fat levels, it is important to recognize their limitations and susceptibility to a high level of operator error that can lead to reporting of erroneously high or low levels of *C. elegans* fat content.

In *C. elegans*, as in mammals, activation of AMPK causes fat reduction [55]. Thus, it may seem paradoxical that loss of *aak-2* could also result in fat reduction. In analogy to mammalian systems, energy deprivation is expected to lead to AMPK activation in *C. elegans* and subsequent mobilization of fat reservoirs as an energy generating strategy. In mammals, the fat reducing effects of AMPK are largely attributable to activation of this kinase complex in peripheral tissues [56]. While it has not been formally shown to be the case in *C. elegans*, we speculate that the noted fat reductions caused by activation of AMPK are similarly dependent on activity of this kinase complex in peripheral tissues. By contrast, our findings indicate that the reduced fat of *aak-2* mutants is due to loss of AAK-2 activity from the nervous system. As in the case of elevated serotonin signaling, inactivation of neural AAK-2 is expected to occur under conditions of plentiful food supplies. In mammals, actions of hormonal cues of food availability on the nervous system are similarly associated with enhanced rates of fat oxidation [57]. For example, increased T3 thyroid hormone, in cases of hyperthyroidism, inactivates hypothalamic AMPK leading to increased brown adipose tissue thermogenesis and weight loss without inducing changes in food intake [58]. Additionally, it has been suggested that hypothalamic inhibition of AMPK may stimulate the sympathetic nervous system that innervates peripheral tissues leading to activation of AMPK in these tissues and stimulation of peripheral fatty acid oxidation [58], [59]. Finally, enhanced serotonin signaling in mammals also promotes enhanced fat oxidation, although it is unknown whether this enhancement is dependent on neural AMPK inhibition [60]–[63]. Thus, in both *C.* elegans and mammals, inhibition of neural AMPK is associated with enhanced peripheral fat oxidation. An area of divergence between *C. elegans* and mammals are the effects of elevated serotonin signaling or inactivation of hypothalamic AMPK on feeding behavior. While elevation of serotonin signaling causes feeding increase through inhibition of AAK-2 in specific neurons of *C. elegans*, elevated serotonin signaling or inhibition of hypothalamic AMPK are thought to cause satiety in mammals [3], [57], [59].

There is ample evidence to assume that rather than simply a binary on/off indicator of food availability, serotonin-signaling functions along a continuum of levels. The available data support the existence of at least three states: a level of serotonin signaling seen in well-fed animals, which is lowered as animals are removed from food, and a highly elevated level that drives transient behaviors such as the enhanced slowing response. One appealing aspect of the regulatory link between serotonin signaling and AAK-2 containing AMPK complexes is that the known features of AMPK regulation could also account for a variety of regulatory states. For instance, AMPK can be in an activated state during periods of nutrient deprivation, in an intermediate state (a non-activated, non-inhibited state) during a well-fed state, or in a fully inhibited state as that mimicked by loss of *aak-2* or elevation of serotonin levels beyond those of well-fed animals. Under standard laboratory conditions, the behavioral phenotypes of *aak-2* mutants, highly elevated feeding rate and dramatically reduced movement rate, are seen transiently when food deprived wild type animals re-encounter food. Therefore, we speculate that the burst of serotonin signaling upon re-encountering food leads to inhibition of neural AAK-2 containing complexes and subsequent behavioral and physiological outcomes seen under these conditions. As serotonin levels return to the level of well-fed animals, neural AAK-2 complexes are likely to be in an intermediate state, neither activated nor fully inhibited.

In numerous organisms, AMPK has been extensively studied as a master regulator of energy balance. In most of these cases, AMPK is considered to function in the context of peripheral tissues and as a downstream effector of hormonal signals [1], [2]. Our findings here demonstrate that the AAK-2 containing AMPK complexes can also act as an upstream regulator of hormonal pathways by modulating their neural secretions. The molecular mechanisms that promote enhanced DCV secretions from the ASI neurons upon AAK-2 inactivation remain to be identified. Whether promotion of DCV secretion is a general feature of AAK-2 inhibition or if it is dependent on particular neural contexts also remains to be seen.

## Materials and Methods

### Strains

Standard *C. elegans* methods were used for strain construction [64]. N2 Bristol was used as the wild type control and the following mutant alleles were analyzed: *ser-5(tm2654)I, aak-1(tm1944)III, tph-1(mg280)III, daf-2(e1370)III, daf-7(e1372)III, unc-31(ft1)IV, aak-2 (ok524)X, tph-1(mg280)III; aak-2(ok524), aak-1(tm1944); aak-2(ok524), daf-2(e1370)III; aak-2(ok524), daf-7(e1372)III; aak-2(ok524), unc-31(ft1)IV; aak-2(ok524)*. Transgenic animals were generated by injecting plasmids and the *unc-122*::*gfp* or *myo-3::gfp* co-injection marker at a concentration of 50 ng/µl. For secretion assays, animals carrying full length DAF-28::mCherry and DAF-7::mCherry driven by a *daf-7* promoter were used. Previously described [41] wild type animals carrying integrated copies of these transgenes were crossed into indicated mutant backgrounds to allow for direct comparisons. Dauer constitutive strains were maintained at 15°C, except when testing dauer entry and maintenance, which were conducted at 25°C.

### Plasmid construction

Plasmids were constructed using Gateway Technology. p*myo-2* and p*myo-3* entry vectors were constructed as described [47]. p*unc-119*::*aak-2a*, p*unc-119*::*aak-2c*, p*grl-21*::*aak-2a*, p*grl-21*::*aak-2c*, p*daf-7*::*aak-2a*, p*daf-7*::*aak-2c* were generated by Gateway cloning. For the *unc-119* promoter, 2000 bp including the ATG was amplified by PCR from genomic DNA and sub-cloned into Gateway entry vector pDONR-P4-P1R. For the *daf-7* promoter, 2800 bp including the ATG was amplified by PCR from genomic DNA and sub-cloned into Gateway entry vector pDONR-P4-P1R. For the *grl-21* promoter, 745 bp including the ATG was amplified by PCR from genomic DNA and sub-cloned into Gateway entry vector pDONR-P4-P1R. Rescue constructs were generated using the pKA453 plasmid to obtain *promoter::orf::intercistronic::GFP* polycistronic fusions. This resulted in the expression of *GFP* from the same transcript as *the ORF* without modification. p*daf-7::daf-7::mCherry* and p*daf-7::daf-28::mCherry*, integrated lines were generated as described in [41]. To generate p*daf-7*::*ser-5*::*gfp*, and p*daf-7::gsa-1*(R182C)::*gfp*, *ser-5* or *gsa-1* cDNA, was amplified and cloned according to the procedure outlined in [7], and recombined using Gateway cloning methodology. The R182C mutation was inserted into the *gsa-1* sequence by oligo-mediated site-directed mutagenesis and the desired mutation was confirmed by sequencing the resulting plasmid.

### Lipid analysis

Extended methods for BODIPY staining, triglyceride measurements, and Sudan Black B staining are provided in reference [65].

Sudan Black B assays were performed at room temperature (22°C) [38] with the following modification to minimize staining variability, which allowed for quantitative comparisons between various genotypes: animals from one genotype were labeled with fluorescein isothiocyanate (FITC) and then fixed and stained in the same tube as unlabeled animals from another genotype. For quantitation, Sudan Black images were collected on a Zeiss Axioplan 2 microscope fitted with a Hamamatsu ORCA-AG camera. Staining intensities were quantitated using Improvision Openlab software. Mean pixel intensity was calculated for staining in the region from the first intestinal cells adjacent to the pharynx midway through the animal to the vulva. Background was determined based on pixel intensity of nonspecific staining in the pharynx. Values are reported as mean pixel intensity minus background for at least ten randomly selected animals per genotype. In each case, test and control animals were fixed and stained in the same tube.

Biochemical determination of extracted triglycerides: synchronized nematodes from a liquid culture of approximately 5000 animals were washed three times by centrifugation and resuspension in 10 ml S-basal medium supplemented with 0.1% PEG-8000. After the washes, the nematode population pellet was finally suspended in approximately 200 µl of S-basal +0.1% PEG-8000. 50 µl of this suspension was reserved for protein determination (see below) and 100 µl of suspension was diluted with 59 µl of water for lipid determination. To extract triglycerides, chloroform (0.2 ml) and methanol (0.4 ml) was added to the 0.159 ml aqueous suspension of nematodes and mixed by periodic vigorous vortexing over 20 min. An additional 0.2 ml of chloroform and 0.2 ml of 0.2 N HCl were then added. The mixture was mixed by vigorous vortexing over 20 min, then centrifuged at 2,500 g for 5 min to separate the phases. The lower phase was washed once with 0.75 ml of the aqueous phase derived from a mixture of chloroform, methanol, 0.1 N HCl (1∶1∶1), then concentrated by vacuum centrifugation. The residue was dissolved in 25 µl of chloroform:methanol (1∶1), 10 µl of which was applied to a thin layer chromatography plate (Merck silica gel-60), along with triglyceride standards (0.5–10 µg). The samples and standards were eluted using a hexanes-ether-acetic acid mixture (70∶30∶1) and the plate was developed by spraying with phosphomolybdic acid stain (Sigma) and heating in a 125°C oven for 10 min. An image of the plate was acquired using a flat bed scanner (Epson) and the integrated density of the bands that exhibited the same elution profile as the triglyceride standards was quantified. The optical densities were then converted to TAG mass by comparison to a TAG standard curve. The mass of lipids obtained from each extraction was normalized to the total protein extractable for 3-4 independent nematode cultures per experimental condition. To determine total protein levels for normalization, 250 µl of extraction buffer (7 M urea, 2 M thiourea, 4% CHAPS, 50 mM tris-HCl pH 7.4, 5 mM TCEP, 1 mM EDTA) was added to the 50 µl suspension of nematodes from the same suspension that was used for TAG extraction. The sample was rotated end-over-end and periodically vortexed for 1 hr at 37°C. The sample was centrifuged (10 min at 16,500 g) and the amount of protein in the supernatant was determined by Bradford assay (Bio-Rad).

For BODIPY staining a 1 mg/ml stock of C_1_-BODIPY 500/512 C_12_ (Invitrogen) was added to NGM plates seeded with OP50 at a final dilution of 1∶50,000. Synchronized L1 animals were added to plates and imaged as Day-1 adults after growth at 20°C. Fluorescent images were acquired on a Zeiss Axioplan II microscope outfitted with a digital CCD camera using the same sub-saturating exposure settings. Using ImageJ, the area surrounding the head of the animal (starting just above the intestinal cells) was selected from which total integrated intensity was derived. At least 10 animals were imaged for each treatment and experiments were repeated a minimum of 2 times. Significance was determined using a student's t-test.

For the coherent anti-stokes Raman scattering, CARS, imaging [32], a picosecond optical parametric oscillator (picoEmerald - APE) with the stokes tuned at 1064 nm and pump at 817 nm in order to match the lipid CH2 stretching mode at 2845 cm^−1^ [33], [34] was used. Both laser pulses are synchronized in time and space and directed to a galvanometric mirror imaging microscope (Nikon Ti-U inverted microscope coupled to a TriM Scope II - LaVision BioTec system) and focused onto the sample by a 60X Nikon objective. The backscattered signal is then directed to a set of dichroic and band pass filters in order to remove the pump and stokes lasers and to detect only the anti-stokes signal in a photomultiplier (Hamamatsu H7422-40). To quantitate the CARS signal for each animal, all of the Z-slices were combined, and background and maximal values were calculated from these combined slices. Regions of interest (head hypodermal region or intestinal areas) were selected and quantitated for each animal and integrated intensity densities are reported for each region. For each genotype and/or serotonin treatment, average data from at least five separate animals are reported.

### 5-HT and fluoxetine treatments

5-HT and fluoxetine treatments were performed as previously described [7], [19].

### Dauer entry and dauer exit

To assess dauer entry, we used 1% SDS treatment as previously describe [40]. Post treatment, animals were gently tapped to monitor movement to determine viability. At least 70 animals per conditions were tested. For dauer exit, 100 animals were plated on each of 5 plates—seeded with OP50 (or HT115 in the case of RNAi bacteria) and rimmed with 40% glycerol—and incubated at the restrictive temperature (25°C). After 48 h, virtually all daf-c animals were in dauer. Plates were kept at 25°C and once per day, animals that had exited dauer were picked off the plates and counted. Graphed data reflects the averages of 5 plates of 100 animals.

### Movement

Well-fed, synchronized young adult animals were washed twice with S-basal and plated on unseeded NGM plates. Movement was assayed 5 min after plating by counting the number of body bends per 20 s. Ten animals were counted per strain.

### Secretion

Strains were synchronized by hypochlorite treatment and the synchronized L1s were plated onto 6 cm NGM plates seeded with OP50. Animals were grown at 20 °C for 2 days until they reached L4 stage. Sub-saturating fluorescence images of the first pair of coelomocytes from 20–30 transgenic animals were recorded at 16× magnification using a Zeiss Axioplan 2 microscope fitted with a Hamamatsu Orca II camera. Fluorescence intensities were quantified using ImageJ software. The outline of each coelomocyte was traced using the image from the p*unc-122*::GFP coelomocyte marker. The fluorescence of DAF-28::mCherry or DAF-7::mCherry within that area was then measured. The mean fluorescence for each cell was subtracted from the minimum fluorescence (background) within that cell. Fluorescence intensities were normalized to the wild type, sham treated control.

### RNAi

HT115 bacteria containing each RNAi vector were tested as previously described. Briefly, bacteria were grown overnight. The following day, bacteria were pelleted and resuspended to 2x concentration prior to plating on NGM agar containing 6 mM IPTG and 25 µg/ml ampicillin. Animals were added to plates (BODIPY staining) or liquid cultures (TLC) as L1s and grown until the L4 stage. Animals were imaged as L4 (BODIPY) or subjected to total lipid extraction followed by thin layer chromatography (TLC).

### Oxygen consumption

Oxygen consumption was measured in synchronized L4 animals that were washed twice with S-basal. Per genotype, 200 animals were placed in a plate with biosensor film used to gauge oxygen consumption (BD Biosciences, Cat# 353830). We previously determined that a three-hour period allows for biosensor film to reach equilibrium. The data are end-point measurements, which reflect oxygen consumption rather than biosensor equilibrium. Per genotype, the measurements were done in quadruplicate, and each experiment repeated 3 times. Measurement of fluorescence was by a Molecular Devices FlexStation.

### Statistical analysis

For pair-wise comparisons, student's t-test was used. For multiple comparisons, one-way ANOVA with Bonferroni correction was used. Error bars represent +/-SEM. *represents statistical difference relative to wild type unless otherwise indicated. P-values are indicated in figure legends.

## Supporting Information

Figure S1 Feeding and movement responses of WT and *aak-2* mutants to increasing doses of exogenous 5-HT. **A.** Treatment of wild type animals with increasing doses of exogenous 5-HT causes a progressive reduction in movement and ultimately paralysis. The movement rates of animals treated with 5 mM 5-HT were similar to those treated with high concentrations of fluoxetine, suggesting that this dose of exogenous 5-HT mimics the effects of elevating endogenously produced 5-HT. Well-fed animals were washed twice with S-Basal and transferred to assay plates without a bacterial lawn. Locomotion rate was recorded after 5 minutes for a minimum of 10 animals of each genotype per concentration. **B.** Relative to wild type animals, *aak-2* mutants are more sensitive to paralysis caused by escalating doses of 5-HT. At least 30 animals of each genotype per concentration were tested. Movement was scored after a 5-minute exposure to 5-HT. **C.** Effects of various doses of 5-HT on feeding. Treatment of WT animals with increasing doses of 5-HT caused a progressive elevation of pumping rate that reached its maximal levels at 5 mM and was not further increased at 10 or 20 mM concentrations of exogenous 5-HT. The already elevated feeding rates of *aak-2* mutants were not further increased by up to 10 mM exogenous 5-HT treatment. Consistent with the enhanced susceptibility of *aak-2* mutants to deleterious effects of high exogenous 5-HT, *aak-2* mutants became sickly and displayed lower than wild type feeding at 20 mM concentration of 5-HT. **D.** 5 mM 5-HT treated wild type animals and untreated *aak-2* animals show significant overlap in transcription expression of indicated metabolic genes relative to untreated wild type animals. Transcript levels of nearly 100 fat and sugar metabolic genes were determined by real-time PCR (RT-PCR). List of the genes and their predicted functions are provided in Table S1. Genes found to be upregulated or downregulated in 5-HT treated wild type animals or in untreated *aak-2* animals were validated using cDNA preparations from two independent nematode growths. Error Bars represent +/−SEM.
(TIF)

Figure S2 **A.** RNAi inactivation of *cpt-1a* (W01A11.5) restored normalized triglycerides (TAG) per protein (TAG/protein) measurement. WT and *aak-2* mutants were grown either on vector RNAi control or on W01A11.5 RNAi. n = 3, *p<0.05, Student's t-test. Error bars represent +/−SEM. **B.** Loss of F08A8.4, encoding a putative acyl-CoA oxidase, via RNAi restores BODIPY staining to *aak-2* mutants. Representative images of BODIPY staining. **C.** Quantitation of hypodermal BODIPY fluorescence intensity shown in B. n = 5, *p<0.05, one-way ANOVA with Bonferroni correction for multiple comparisons.
(TIF)

Figure S3 Transcript levels of *tph-1* and *aak-*1 are unchanged in *aak-2* mutants relative to WT as assessed by RT-PCR assay. In each case, data are normalized to average of the WT levels. n = 3, error bars represent +/−SEM.
(TIF)

Figure S4 Quantitation of BODIPY fluorescence intensity shows that reconstitution of *aak-2* in various peripheral tissues or in neurons implicated in feeding or dauer exit does not rescue the low fat of *aak-2* mutants. Data for reconstitution in the body wall muscle (*pmyo-3::aak-2*), hypodermis (*pgrl-21::aak-2*), *hlh-34* neurons, likely AVJ, implicated in feeding elevation upon AAK-2 inactivation (*phlh-34::aak-2*), ASI neurons (*pdaf-7::aak-2*) are shown. n = 10, *p<0.05, one-way ANOVA with Bonferroni correction for multiple comparisons. Asterisks indicate significance relative to WT.
(TIF)

Figure S5 *aak-2* deficient dauer animals have reduced fat at all stages. **A-D.** Sudan Black B staining of three representative *daf-7* (A,C) and *daf-7; aak-2* (B,D) animals on day 1 (A,B) and day 4 (C,D) of dauer. **E-H.** Representative BODIPY-labeled fatty acid staining of *daf-7* (E,G) and *daf-7; aak-2* (F,H) as L2 larvae (E,F) and L4 larvae (G,H). **I-J**. Representative images of BODIPY-labeled fatty acid stained *daf-2* (I) and *daf-2; aak-2* (J) L4 larvae. **K-N.** Sudan Black B staining of representative *daf-2* (K, M) and *daf-7; aak-2* (L,N) animals on day 1 (K,L) and day 4 (M,N) of dauer.
(TIF)

Figure S6 *daf-2; aak-2* animals have reduced fat relative to *daf-2* animals. Quantitation of signal intensities of coherent anti-Stokes Raman Scattering, CARS, imaging of *daf-2* and *daf-2*; *aak-2* as L4 animals +/− 5 mM 5-HT treatment. 5-HT treatment lowered the CARS signal intensities from head hypodermal regions of *daf-2* animals. *daf-2*; *aak-2* mutants had lower signal intensities relative to *daf-2*, which was not further reduced by 5 mM 5-HT treatment. Signal intensities of 5-HT treated *daf-2* were not significantly different than those of *aak-2* +/− 5-HT. n = 5, *p<0.05, Student's t-test.
(TIF)

Figure S7 **A.** As in DAF-28::mCherry (Figure 4), treatment with 5 mM 5-HT or loss of *aak-2* promote enhanced secretions of DAF-7::mCherry expressed in the ASI neurons using a *daf-7* promoter, as assessed by the coelomocyte accumulation assay. p*unc-122*::*GFP* was used to mark coelomocytes. The *daf-7::mCherry* transgene was introduced into indicated backgrounds by crossing. WT and *aak-2* mutants were sham treated. Data are shown relative to sham treated WT animals. Each bar represents examination of 20–30 transgenic animals. Error bars represent standard error. *p<0.05, one-way ANOVA with Bonferroni correction for multiple comparisons. **B.** Loss of *aak-2* does not significantly alter gene expressions of *daf-7, daf-28*, or *ser-5* as measured by RT-PCR. In each case, data are normalized to average of the WT levels. n = 3, error bars represent +/−SEM **C.** Loss of *unc-31* abrogates the elevated coelomocyte accumulation of DAF-28::mCherry seen upon 5 mM 5-HT treatment or loss of *aak-2*. Error bars represent standard error. Asterisks denotes significance relative to sham treated WT animals, *p<0.05, one-way ANOVA with Bonferroni correction for multiple comparisons. An average of 20–30 transgenic animals were examined for each bar. The *daf-28* neuropeptide was tagged with mCherry, while p*unc-122*::*GFP* was used to mark coelomocytes.
(TIF)

Figure S8 Model. Inactivation of AAK-2 in the *hlh-34* expressing AVJ neurons mediate the effects of elevated serotonin signaling on feeding while inactivation of AAK-2 in the ASI neurons mediates the effects of elevated serotonin signaling in enhanced release of the DAF-7 TGF-β ligand and the DAF-28 insulin. In both cases, serotonin signaling through the SER-5 receptor leads to activation of Protein Kinase A, which in turn, causes inhibition of AAK-2.
(TIF)

Table S1 Effects of 5 mM 5-HT and *aak-2* loss on expression of select metabolic genes. Predicted annotations for each of the genes are indicated. RT-PCR assays were repeated at least twice for genes whose transcription was altered by addition of 5 mM 5-HT or loss of *aak-2*. Data are normalized to untreated WT for each gene.
(DOCX)
