# Supplementary figures and images for: Loss of a Neural AMP-Activated Kinase Mimics the Effects of Elevated Serotonin on Fat, Movement, and Hormonal Secretions
Source: PLoS Genet. 2014 Jun 12;10(6):e1004394. doi: 10.1371/journal.pgen.1004394 (PMC4055570; doi:10.1371/journal.pgen.1004394)

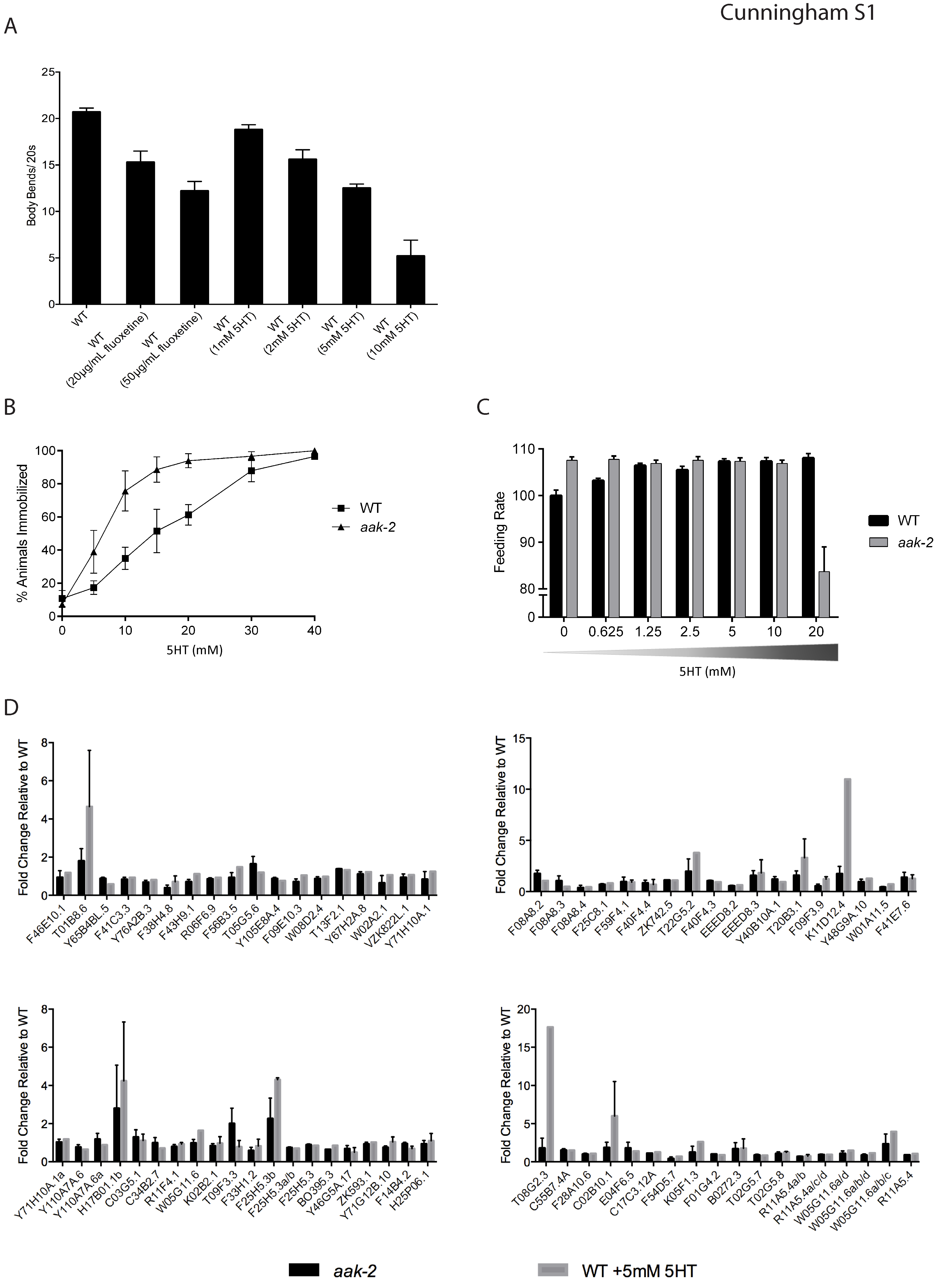

Supplement: Figure S1 — Feeding and movement responses of WT and aak-2 mutants to increasing doses of exogenous 5-HT. A. Treatment of wild type animals with increasing doses of exogenous 5-HT causes a progressive reduction in movement and ultimately paralysis. The movement rates of animals treated with 5 mM 5-HT were similar to those treated with high concentrations of fluoxetine, suggesting that this dose of exogenous 5-HT mimics the effects of elevating endogenously produced 5-HT. Well-fed animals were washed twice with S-Basal and transferred to assay plates without a bacterial lawn. Locomotion rate was recorded after 5 minutes for a minimum of 10 animals of each genotype per concentration. B. Relative to wild type animals, aak-2 mutants are more sensitive to paralysis caused by escalating doses of 5-HT. At least 30 animals of each genotype per concentration were tested. Movement was scored after a 5-minute exposure to 5-HT. C. Effects of various doses of 5-HT on feeding. Treatment of WT animals with increasing doses of 5-HT caused a progressive elevation of pumping rate that reached its maximal levels at 5 mM and was not further increased at 10 or 20 mM concentrations of exogenous 5-HT. The already elevated feeding rates of aak-2 mutants were not further increased by up to 10 mM exogenous 5-HT treatment. Consistent with the enhanced susceptibility of aak-2 mutants to deleterious effects of high exogenous 5-HT, aak-2 mutants became sickly and displayed lower than wild type feeding at 20 mM concentration of 5-HT. D. 5 mM 5-HT treated wild type animals and untreated aak-2 animals show significant overlap in transcription expression of indicated metabolic genes relative to untreated wild type animals. Transcript levels of nearly 100 fat and sugar metabolic genes were determined by real-time PCR (RT-PCR). List of the genes and their predicted functions are provided in Table S1. Genes found to be upregulated or downregulated in 5-HT treated wild type animals or in untreated aak-2 animals wer [file pgen.1004394.s001.tif]

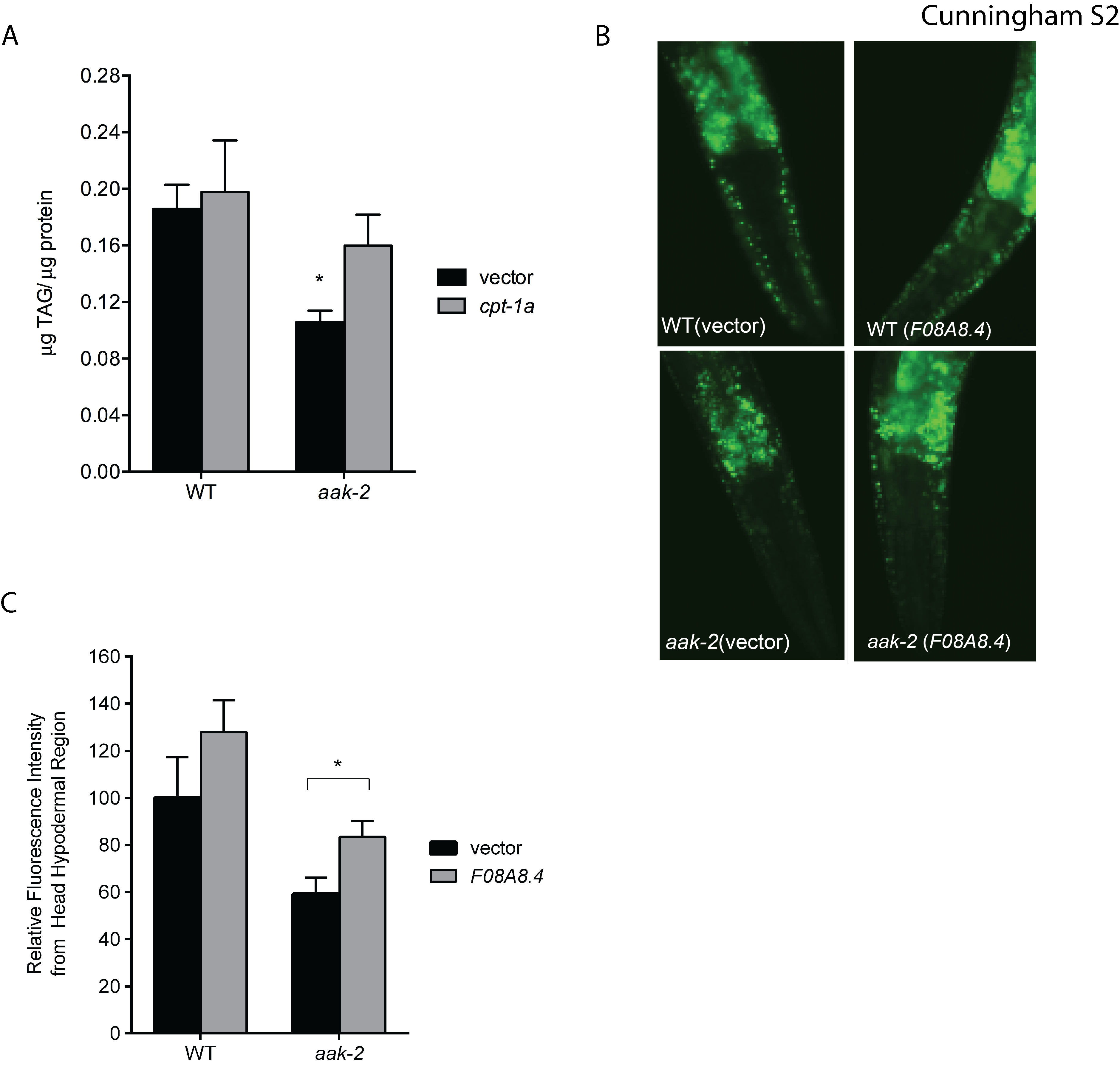

Supplement: Figure S2 — A. RNAi inactivation of cpt-1a (W01A11.5) restored normalized triglycerides (TAG) per protein (TAG/protein) measurement. WT and aak-2 mutants were grown either on vector RNAi control or on W01A11.5 RNAi. n = 3, *p<0.05, Student's t-test. Error bars represent +/−SEM. B. Loss of F08A8.4, encoding a putative acyl-CoA oxidase, via RNAi restores BODIPY staining to aak-2 mutants. Representative images of BODIPY staining. C. Quantitation of hypodermal BODIPY fluorescence intensity shown in B. n = 5, *p<0.05, one-way ANOVA with Bonferroni correction for multiple comparisons. (TIF) [file pgen.1004394.s002.tif]

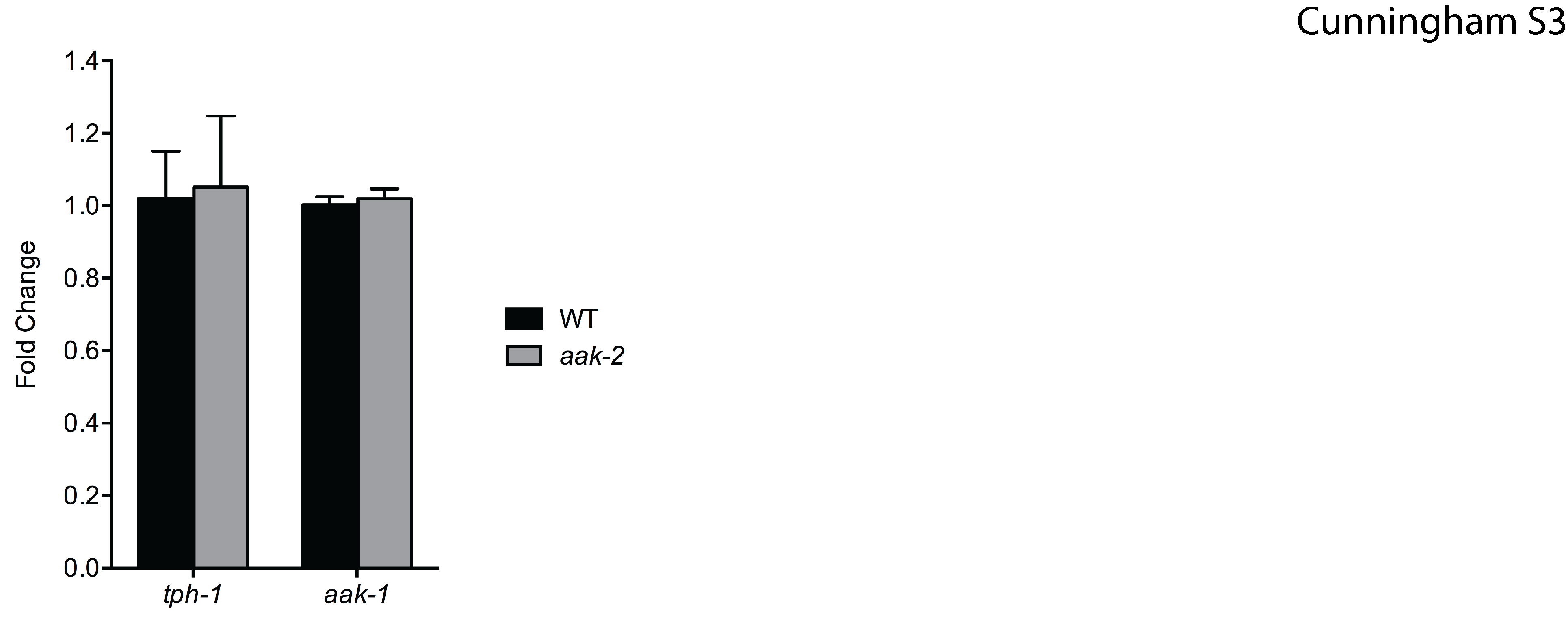

Supplement: Figure S3 — Transcript levels of tph-1 and aak-1 are unchanged in aak-2 mutants relative to WT as assessed by RT-PCR assay. In each case, data are normalized to average of the WT levels. n = 3, error bars represent +/−SEM. (TIF) [file pgen.1004394.s003.tif]

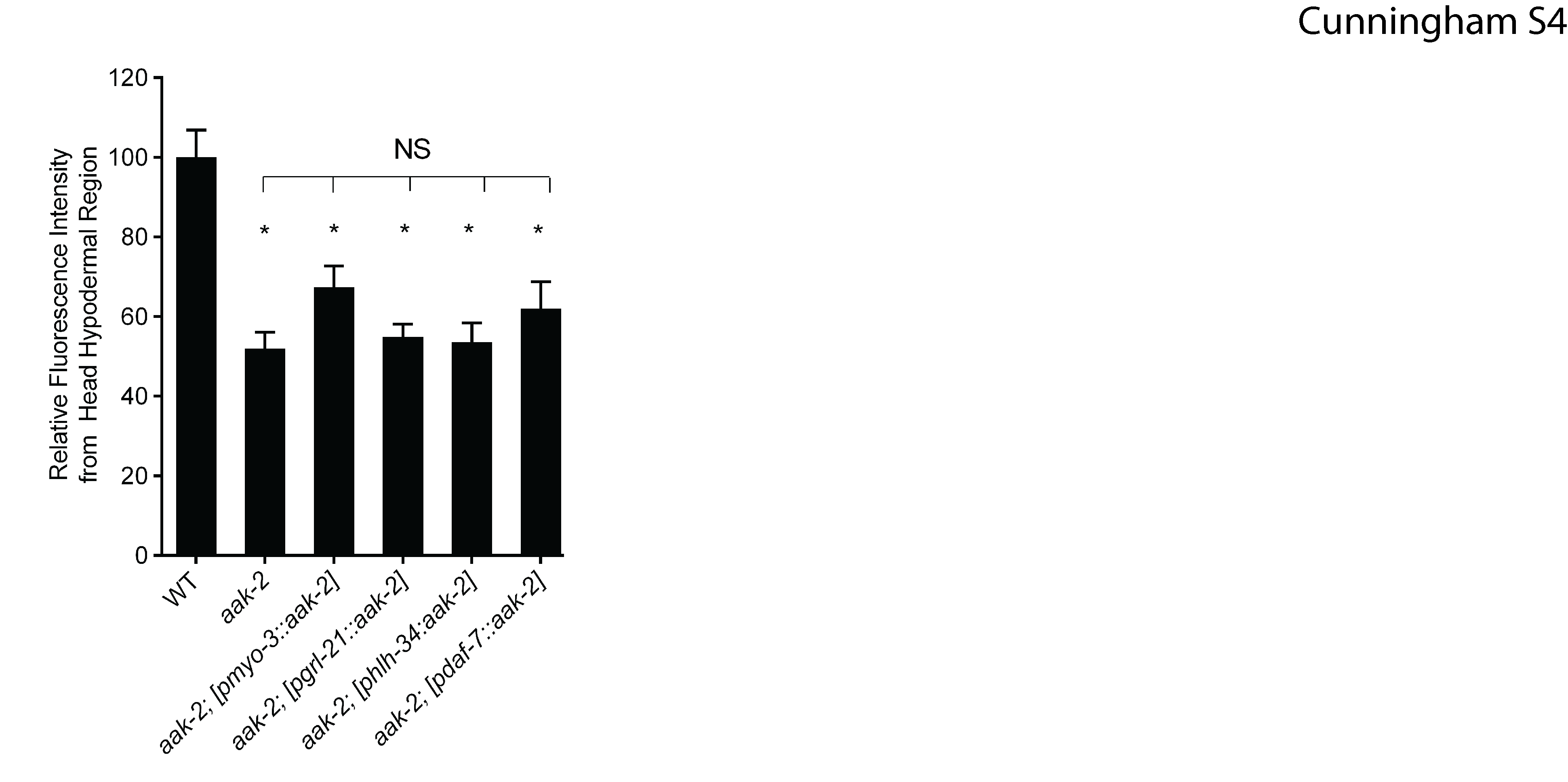

Supplement: Figure S4 — Quantitation of BODIPY fluorescence intensity shows that reconstitution of aak-2 in various peripheral tissues or in neurons implicated in feeding or dauer exit does not rescue the low fat of aak-2 mutants. Data for reconstitution in the body wall muscle (pmyo-3::aak-2), hypodermis (pgrl-21::aak-2), hlh-34 neurons, likely AVJ, implicated in feeding elevation upon AAK-2 inactivation (phlh-34::aak-2), ASI neurons (pdaf-7::aak-2) are shown. n = 10, *p<0.05, one-way ANOVA with Bonferroni correction for multiple comparisons. Asterisks indicate significance relative to WT. (TIF) [file pgen.1004394.s004.tif]

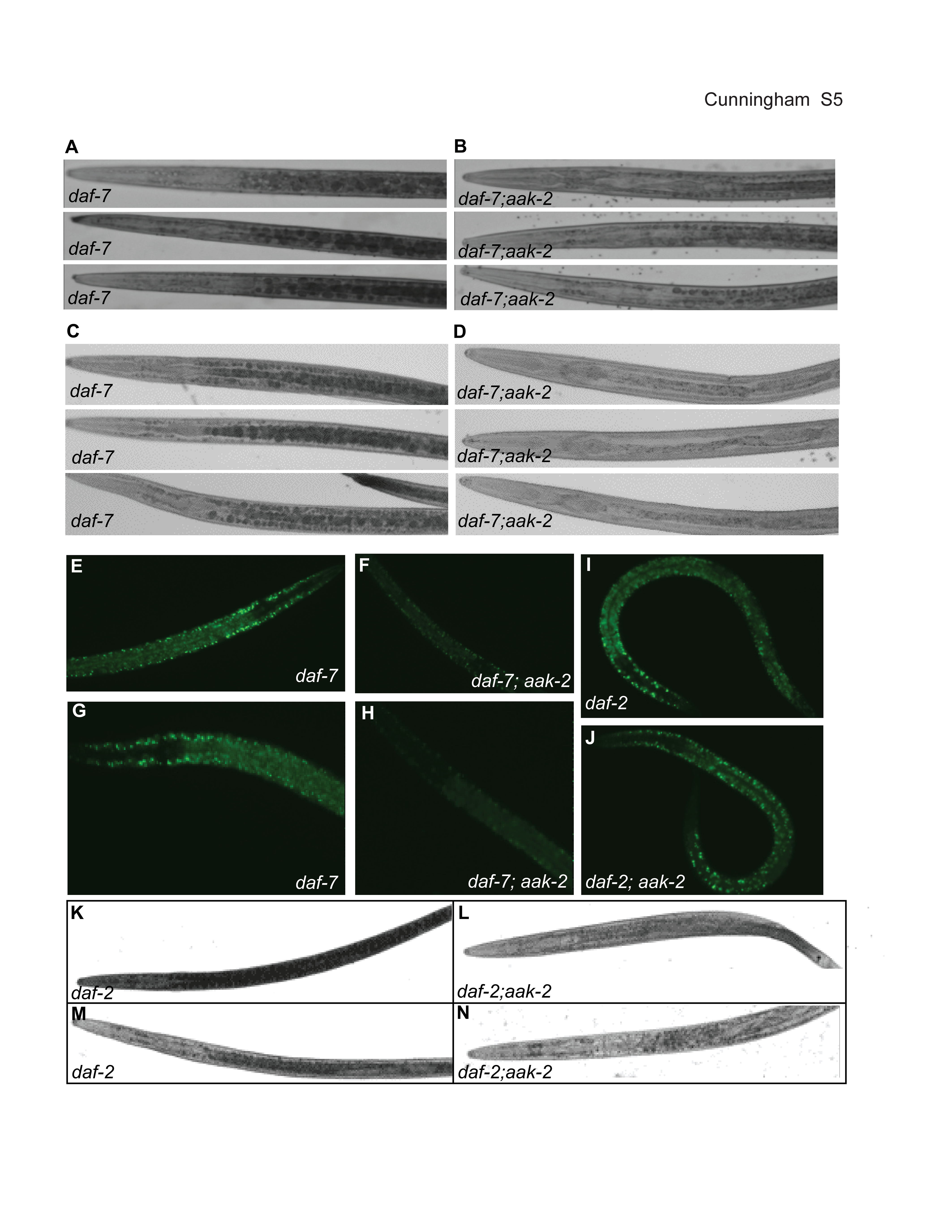

Supplement: Figure S5 — aak-2 deficient dauer animals have reduced fat at all stages. A-D. Sudan Black B staining of three representative daf-7 (A,C) and daf-7; aak-2 (B,D) animals on day 1 (A,B) and day 4 (C,D) of dauer. E-H. Representative BODIPY-labeled fatty acid staining of daf-7 (E,G) and daf-7; aak-2 (F,H) as L2 larvae (E,F) and L4 larvae (G,H). I-J. Representative images of BODIPY-labeled fatty acid stained daf-2 (I) and daf-2; aak-2 (J) L4 larvae. K-N. Sudan Black B staining of representative daf-2 (K, M) and daf-7; aak-2 (L,N) animals on day 1 (K,L) and day 4 (M,N) of dauer. (TIF) [file pgen.1004394.s005.tif]

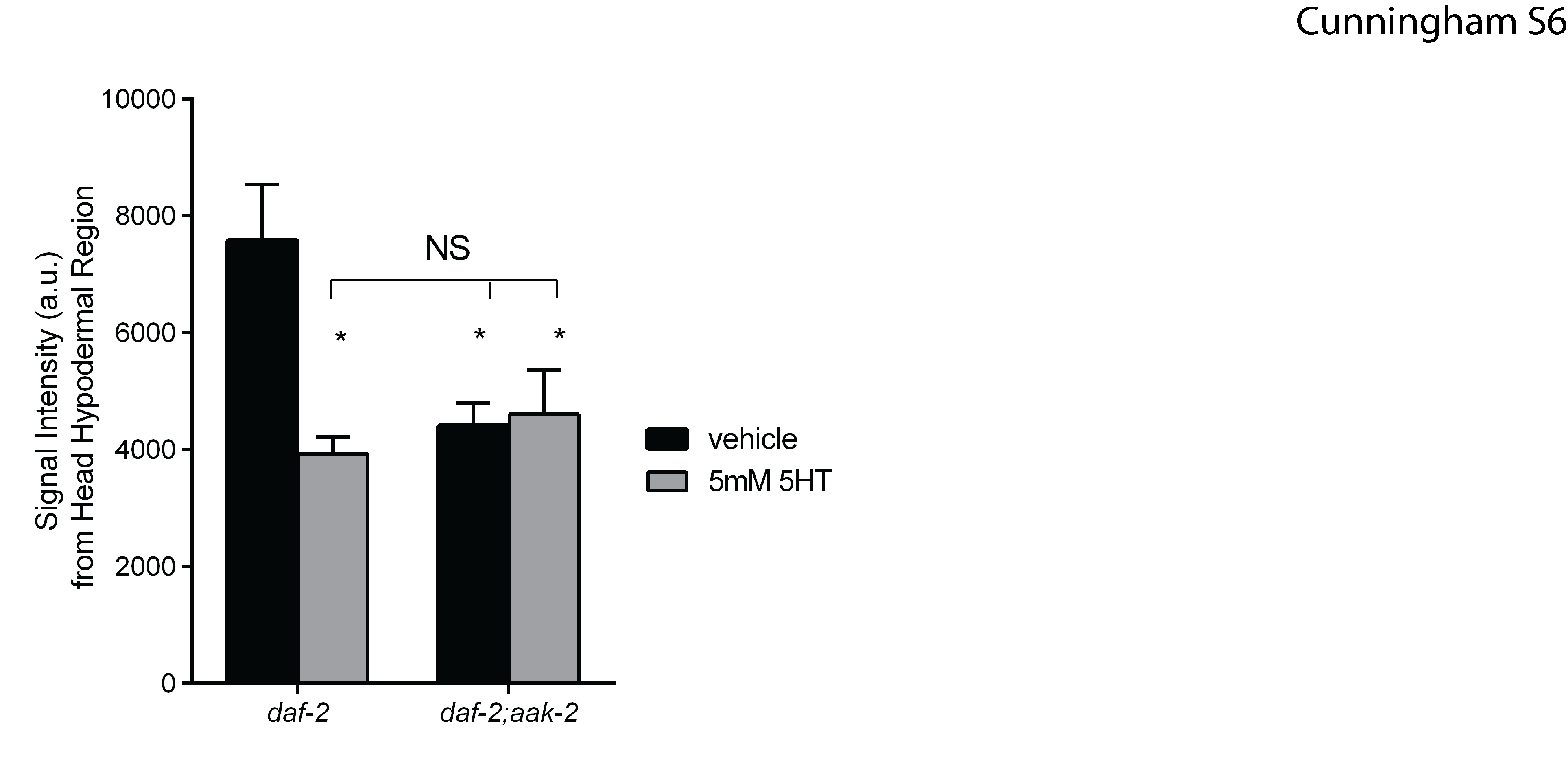

Supplement: Figure S6 — daf-2; aak-2 animals have reduced fat relative to daf-2 animals. Quantitation of signal intensities of coherent anti-Stokes Raman Scattering, CARS, imaging of daf-2 and daf-2; aak-2 as L4 animals +/− 5 mM 5-HT treatment. 5-HT treatment lowered the CARS signal intensities from head hypodermal regions of daf-2 animals. daf-2; aak-2 mutants had lower signal intensities relative to daf-2, which was not further reduced by 5 mM 5-HT treatment. Signal intensities of 5-HT treated daf-2 were not significantly different than those of aak-2 +/− 5-HT. n = 5, *p<0.05, Student's t-test. (TIF) [file pgen.1004394.s006.tif]

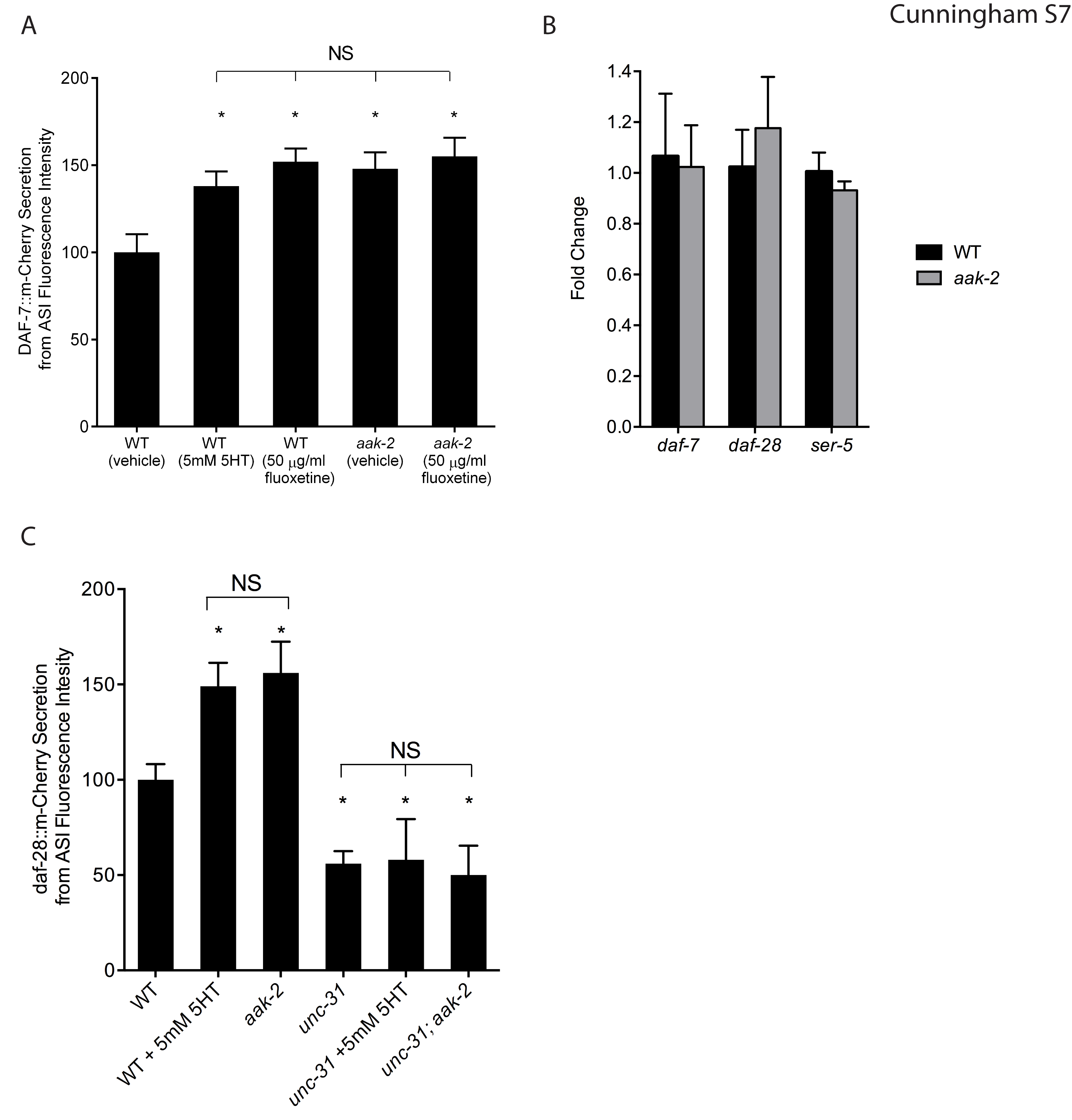

Supplement: Figure S7 — A. As in DAF-28::mCherry (Figure 4), treatment with 5 mM 5-HT or loss of aak-2 promote enhanced secretions of DAF-7::mCherry expressed in the ASI neurons using a daf-7 promoter, as assessed by the coelomocyte accumulation assay. punc-122::GFP was used to mark coelomocytes. The daf-7::mCherry transgene was introduced into indicated backgrounds by crossing. WT and aak-2 mutants were sham treated. Data are shown relative to sham treated WT animals. Each bar represents examination of 20–30 transgenic animals. Error bars represent standard error. *p<0.05, one-way ANOVA with Bonferroni correction for multiple comparisons. B. Loss of aak-2 does not significantly alter gene expressions of daf-7, daf-28, or ser-5 as measured by RT-PCR. In each case, data are normalized to average of the WT levels. n = 3, error bars represent +/−SEM C. Loss of unc-31 abrogates the elevated coelomocyte accumulation of DAF-28::mCherry seen upon 5 mM 5-HT treatment or loss of aak-2. Error bars represent standard error. Asterisks denotes significance relative to sham treated WT animals, *p<0.05, one-way ANOVA with Bonferroni correction for multiple comparisons. An average of 20–30 transgenic animals were examined for each bar. The daf-28 neuropeptide was tagged with mCherry, while punc-122::GFP was used to mark coelomocytes. (TIF) [file pgen.1004394.s007.tif]

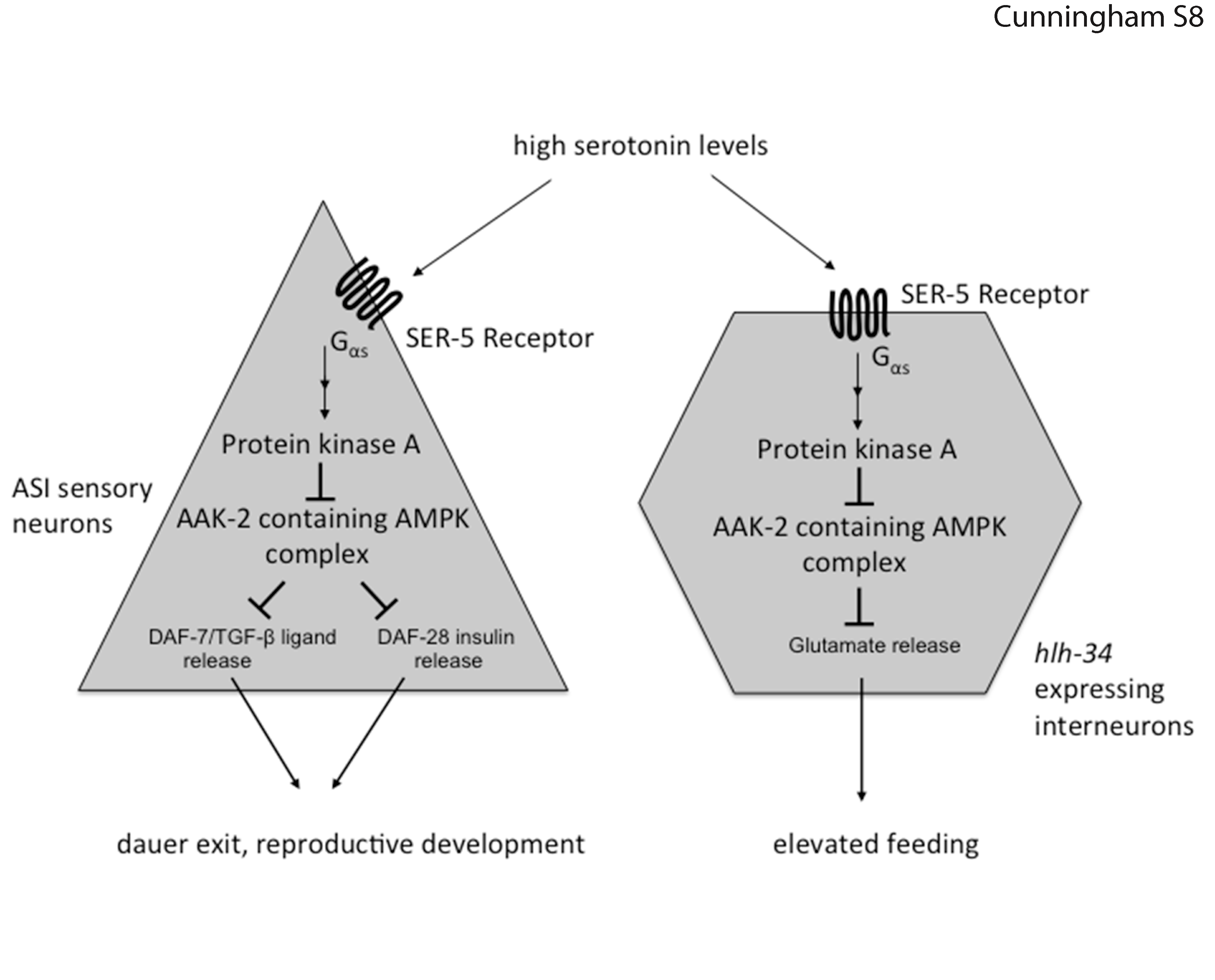

Supplement: Figure S8 — Model. Inactivation of AAK-2 in the hlh-34 expressing AVJ neurons mediate the effects of elevated serotonin signaling on feeding while inactivation of AAK-2 in the ASI neurons mediates the effects of elevated serotonin signaling in enhanced release of the DAF-7 TGF-β ligand and the DAF-28 insulin. In both cases, serotonin signaling through the SER-5 receptor leads to activation of Protein Kinase A, which in turn, causes inhibition of AAK-2. (TIF) [file pgen.1004394.s008.tif]
