# Supplementary material for: Loss of a Neural AMP-Activated Kinase Mimics the Effects of Elevated Serotonin on Fat, Movement, and Hormonal Secretions
Source: PLoS Genet. 2014 Jun 12;10(6):e1004394. doi: 10.1371/journal.pgen.1004394 (PMC4055570; doi:10.1371/journal.pgen.1004394)
Supplement: Table S1 — Effects of 5 mM 5-HT and aak-2 loss on expression of select metabolic genes. Predicted annotations for each of the genes are indicated. RT-PCR assays were repeated at least twice for genes whose transcription was altered by addition of 5 mM 5-HT or loss of aak-2. Data are normalized to untreated WT for each gene. (DOCX) [file pgen.1004394.s009.docx]

| Supplementary Table 1. Effects of 5mM 5HT and *aak-2* loss on expression of select metabolic genes | | | |
| --- | --- | --- | --- |
| Gene ID |  | **WT + 5mM 5HT** | ***aak-2*** |
| FAT METABOLISM | | | |
| F46E10.1 | Acyl-CoA Synthetase | 1.18 | 0.93 ± 0.35 |
| T01B8.6 | Acyl-CoA Synthetase | 4.64 ± 2.94 | 1.80 ± 0.64 |
| Y65B4BL.5 | Acyl-CoA Synthetase | 0.59 | 0.88 ± 0.06 |
| Y76A2B.3 | Acyl-CoA Synthetase | 0.81 | 0.68 ± 0.10 |
| F38H4.8 | Enoyl-CoA Hydratase | 0.70 ± 0.30 | 0.38 ± 0.15 |
| F43H9.1 | Enoyl-CoA Hydratase | 1.12 | 0.71 ± 0.11 |
| R06F6.9 | Enoyl-CoA Hydratase | 0.92 | 0.85 ± 0.06 |
| F56B3.5 | Enoyl-CoA Hydratase | 1.48 | 0.93 ± 0.25 |
| T05G5.6 | Enoyl-CoA Hydratase | 1.20 | 1.64 ± 0.38 |
| Y105E8A.4 | Enoyl-CoA Hydratase | 0.76 | 0.87 ± 0.061 |
| F09E10.3 | Short-chain Dehydrogenase | 1.05 | 0.71 ± 0.14 |
| K05F1.3 | Acyl-CoA Dehydrogenase | 2.63 | 1.26 ± 0.77 |
| F01G4.2 | 3OH-Acyl CoA Dehydrogenase | 0.89 | 1.01 ± 0.02 |
| B0272.3 | 3OH-Acyl CoA Dehydrogenase | 1.7600 ± 1.24 | 1.71 ± 0.79 |
| F08A8.2 | Acyl-CoA Oxidase-1 | 1.06 | 1.77 ± 0.33 |
| F08A8.3 | Acyl-CoA Oxidase | 0.49 | 1.05 ± 0.49 |
| F08A8.4 | Acyl-CoA Oxidase | 0.46 | 0.38 ± 0.24 |
| F25C8.1 | Acyl-CoA Oxidase-1 | 0.83 | 0.71 ±0.08 |
| F59F4.1 | Acyl-CoA Oxidase-1 | 0.93 ± 0.20 | 0.96 ±0.46 |
| T02G5.4 | Acetyl-CoA Thiolase | 0.92 | 1.11 ± 0.01 |
| B0303.3 | KetoAcyl-CoA Thiolase | 1.50 | 1.22 ± 0.02 |
| W08D2.4 | Fat-3 Δ6 Desaturase | 0.98 | 0.87 ± 0.10 |
| T13F2.1 | Fat-4 Δ5 Desaturase | 1.33 | 1.37 ± 0.03 |
| Y67H2A.8 | Fat-1 3 Desaturase | 1.22 | 1.11 ± 0.12 |
| W02A2.1 | Fat-2 Δ12 Desaturase | 1.07 | 0.64 ± 0.39 |
| VZK822L.1 | Fat-6 | 1.07 | 0.93 ± 0.18 |
| ZK742.5 | lbp-6 | 0.69 ± 0.51 | 0.83 ± 0.29 |
| T22G5.2 | lbp-7 | 1.12 | 1.13 ± 0.01 |
| T22G5.6 | lbp-8 | 3.79 | 1.98 ± 1.23 |
| F40F4.3 | lbp-1 | 0.94 | 1.06 ± 0.07 |
| EEED8.2 | Fatty Acid Binding Protein | 0.64 | 0.57 ± 0.06 |
| EEED8.3 | Fatty Acid Binding Protein | 1.83 ± 1.28 | 1.56 ± 0.48 |
| Y40B10A.1 | Fatty Acid Binding Protein | 0.96 | 1.20 ± 0.28 |
| C07E3.9 | Phospholipase A2 | 0.86 | 1.03 ± 0.13 |
| C42D8.5 | Angiotensin Conv Enzy | 1.09 | 0.68 ± 0.38 |
| C44B7.8 | ATP Binding Protein | 0.85 | 0.64 ± 0.01 |
| C25A1.5 | Fatty-Acid Hydroxylase | 0.50 ± 0.25 | 0.68 ± 0.18 |
| C44B7.9 | Peroxisomal Membrane | 2.12 | 0.82 ± 0.13 |
| Y56A3A.19 | Acyl Carrier Protein | 0.97 | 0.86 ± 0.10 |
| T02G5.7 | Acetyl-CoA Acetyltransferase | 0.84 | 0.88 ± 0.12 |
| T02G5.8 | Acetyl-CoA Acetyltransferase | 1.24 ± 0.12 | 1.07 ± 0.22 |
| C50D2.7 | GlucoKinase | 0.98 | 0.97 ± 0.22 |
| T08G2.3 | Acyl-CoA Dehydrogenase | 17.64 | 1.79 ± 1.31 |
| C55B7.4A | Acyl-CoA Dehydrogenase | 1.51 | 1.53 ± 0.19 |
| F28A10.6 | Acyl-CoA Dehydrogenase | 1.08 | 1.01 ± 0.11 |
| C02B10.1 | Acyl-CoA Dehydrogenase | 5.99 ± 4.55 | 1.86 ± 0.69 |
| E04F6.5 | Acyl-CoA Dehydrogenase | 1.39 | 1.79 ± 0.76 |
| C17C3.12A | Acyl-CoA Dehydrogenase | 1.27 | 1.10 ± 0.01 |
| F54D5.7 | Acyl-CoA Dehydrogenase | 0.70 | 0.40 ± 0.23 |
| F41C3.3 | Acyl-CoA Synthase | 0.93 | 0.82 ± 0.11 |
| T20B3.1 | Carnitine Palmitoyl Transferase | 3.32 ± 1.84 | 1.58 ± 0.46 |
| F09F3.9 | Carnitine Palmitoyl Transferase | 1.22 ± 0.25 | 0.55 ± 0.18 |
| K11D12.4 | Carnitine Palmitoyl Transferase | 10.98 | 1.74 ± 0.72 |
| Y48G9A.10 | Carnitine Palmitoyl Transferase | 1.30 | 0.95 ± 0.26 |
| W01A11.5 | Carnitine Palmitoyl Transferase | 0.74 | 0.46 ± 0.05 |
| F41E7.6 | Carnitine Palmitoyl Transferase | 1.28 ± 0.38 | 1.39 ± 0.50 |
| CARBOHYDRATE METABOLISM | | | |
| C05C10.3 | Succinyl-CoA;3-ketoacid CoA Transferase | 0.95 | 0.75 ± 0.11 |
| F25B4.6 | HMG-CoA Synthase | 0.98 | 0.78 ± 0.01 |
| Y71G12B.10 | HMG-CoA Lyase | 1.04 ± 0.26 | 0.75 ± 0.07 |
| F14B4.2 | Hexokinase | 0.69 ± 0.13 | 0.94 ± 0.07 |
| H25P06.1 | Hexokinase | 1.09 ± 0.39 | 0.93 ± 0.17 |
| Y71H10A.1 | 6-Phosphofructokinase (both spliceforms) | 1.25 | 0.83 ± 0.41 |
| Y71H10A.1a | 6-phosphofructokinase (a spliceform) | 1.18 | 1.03 ± 0.14 |
| R11A5.4 | PEPCK (all spliceforms) | 1.06 | 0.89 ± 0.02 |
| W05G11.6 | PEPCK (all spliceforms) | 1.64 | 0.98 ± 0.19 |
| F25H5.3 | Pyruvate Kinase (all spliceforms) | 0.85 | 0.89 ± 0.03 |
| ZK593.1 | Pyruvate Kinase | 1.02 | 0.93 ± 0.09 |
| Y110A7A.6 | Phosphofructokinase (both spliceforms) | 0.65 | 0.77 ± 0.12 |
| K02B2.1 | Phosphofructokinase | 0.97 ± 0.34 | 0.83 ± 0.11 |
| T09F3.3 | Glyceraldehyde-3 Phosphate Dehydroge | 0.78 ± 0.32 | 2.0 ± 0.81 |
| F33H1.2 | Glyceraldehyde-3 Phosphate Dehydroge | 0.82 ± 0.35 | 0.59 ± 0.17 |
| R11A5.4a/b | PEPCK (a and b spliceforms) | 0.76 ± 0.19 | 0.69 ± 0.08 |
| R11A5.4a/c/d | PEPCK (a, c and d isoforms) | 0.96 | 0.94 ± 0.08 |
| W05G11.6a/d | PEPCK (a and d spliceforms) | 1.49 | 1.08 ± 0.33 |
| W05G11.6a/b/d | PEPCK (a,b and d spliceforms) | 1.15 | 0.87 ± 0.17 |
| W05G11.6a/b/c | PEPCK (a,b and c spliceforms) | 3.95 | 2.34 ± 1.29 |
| F25H5.3b | Pyruvate Kinase (b spliceform) | 4.31 ± 0.10 | 2.26 ± 1.07 |
| F25H5.3a/b | Pyruvate Kinase (a and b spliceforms) | 0.70 | 0.75 ± 0.04 |
| Y110A7A.6a | Phosphofructokinase (a isoform) | 0.88 | 1.19 ± 0.30 |
| H17B01.1b | Sugar Transporter (b isoform) | 4.24 ± 3.09 | 2.80 ± 2.25 |
| K07A3.1 | fructose, 1,6 bisphosphatase | 1.14 | 0.83 ± 0.20 |
| F54H12.1a/b | aconitase (a and b spliceforms) | 0.81 | 0.80 ± 0.07 |
| F54H12.1a/b/c | aconitase (a, b and c spliceforms) | 1.10 | 0.87 ± 0.01 |
| ZK455.1 | aconitase | 1.07 | 0.89 ± 0.17 |
| F20H11.3 | malate dehydrogenase | 1.06 | 0.98 ± 0.21 |
| F46E10.10a/c | lactate/malate dehydrogenase (a and c) | 0.73 | 0.99 ± 0.27 |
| F46E10.10a/b | lactate/malate dehydrogenase (a and b) | 0.77 | 0.86 ± 0.07 |
| C05E4.9.a | Isocitrate lyase family/Malate synthase | 1.53 | 1.09 ± 0.39 |
| C05E4.9.b | Isocitrate lyase family/Malate synthase | 1.23 | 1.18 ± 0.51 |
| C03G5.1 | succinate dehydrogenase | 1.11 ± 0.33 | 1.29 ± 0.38 |
| C34B2.7 | succinate dehydrogenase | 0.71 | 0.98 ± 0.27 |
| R11F4.1 | glycerol kinase | 0.93 ± 0.08 | 0.79 ± 0.10 |

**­­­­­**
